# Trajectory patterns of blood pressure change up to six years and the risk of dementia: a nationwide cohort study

**DOI:** 10.18632/aging.203228

**Published:** 2021-07-01

**Authors:** Gang Cheng, Simin He, Qiong He, Xiaowei Xie, Cai Tang, Qunhui Xie, Xihong Wu, Ni Jiang, Chao Li, Xianying Min, Yan Yan

**Affiliations:** 1 Department of Epidemiology and Health Statistics, Xiangya School of Public Health, Central South University, Changsha, Hunan, China

**Keywords:** blood pressure, trajectory, dementia, pulse pressure, late-life

## Abstract

The present study aimed to investigate the associations between the trajectory of blood pressure (BP) change and the risk of subsequent dementia and to explore the differences in age, gender, and hypertension subgroups. We included 10,660 participants aged ≥ 60 years from 1998 to 2018 waves of the Chinese Longitudinal Healthy Longevity Survey. Latent growth mixture models were used to estimate BP trajectories. Cox-proportional hazard models were used to analyze the effects of BP trajectories on the risk of dementia. According to the results, stabilized systolic BP (SBP) was found to be associated with a higher risk of dementia compared with normal SBP [adjusted hazard ratio (aHR): 1.62; 95% confidence interval (CI): 1.27-2.07] and elevated SBP (aHR: 2.22; 95% CI: 1.51-3.28) in and only in the subgroups of the oldest-old, women, and subjects without hypertension at baseline. Similarly, stabilized pulse pressure (PP) was associated with a higher risk of dementia compared with normal PP (aHR: 1.52; 95% CI: 1.24-1.88) and elevated PP (aHR: 2.12; 95% CI: 1.48-3.04) in and only in the subgroups of the oldest-old, women, and subjects with hypertension at baseline. These findings suggest that stabilized SBP and PP have predictive significance for the occurrence of dementia in late life, and the factors of age, gender, and late-life hypertension should be considered when estimating the risk of BP decline on dementia.

## INTRODUCTION

Dementia is a common public health challenge worldwide, affecting nearly 47 million people in 2015, and this figure is expected to rise to 131 million by 2050 [1]. In view of the lack of effective treatment on cognitive decline for patients with dementia and the poor prognosis of progressive exacerbation, exploring possible preventive measures to reduce the prevalence of dementia or delay its onset may be a reasonable clinical strategy with important practical significance [2]. Given this, vascular factors have aroused an increasing interest from researchers, while controlling these factors provides a feasible method to prevent cerebrovascular impairment [2].

Blood pressure (BP) as one of the modifiable vascular factors was observed to be associated with dementia outcomes in various forms, such as mean measure [3], prehypertension [4], orthostatic hypotension [5], and pulse pressure (PP) [6]. Recently, accumulated evidence indicates that BP variability, which contributes to cerebrovascular damage as an independent factor of mean BP [7], is positively associated with the risk of dementia [2, 8–11]. The possible mechanism may be that a higher BP variability is correlated with increased hyperintensity lesions of white matter on brain imaging [12], increased intima-media thickness of carotid artery [13], and accelerated progression of early carotid atherosclerosis [2, 14]. However, the patterns of BP change over a certain period, which cannot be described by BP variability or any single measure, have not been comprehensively elucidated. BP trajectory is helpful for capturing the dynamic change of BP within a certain period and visualizing the direction and size of BP variability. Recent longitudinal studies suggest that subgroups with distinct trajectories of BP change may be associated with a variety of clinical outcomes such as cardiovascular events [15–18], stroke [18, 19], and all-cause mortality [17, 18, 20]. Given the close association between the cardiovascular and cerebrovascular burden and dementia [21, 22], we hypothesized that BP trajectory patterns would have predictive significance for the risk of subsequent dementia outcomes.

At present, limited results have been reported regarding the effect of BP change trajectory on incident dementia. Most of the existing studies were focused on the effects of longitudinal measurement, baseline value, or BP decline on the risk of subsequent dementia outcomes, and BP trajectory was consistently described across a number of studies based on whether the aged adults were diagnosed of dementia in a later time [23–29]. By following up with 707 middle-aged women for 37 years, the Prospective Population Study of Women in Gothenburg reported that subjects who developed dementia had a steeper decline in systolic BP (SBP) during the five years before diagnosis than those who did not [24]. Another 32-year cohort study (*n*=1,890) observed that male subjects who developed dementia had a greater increase in SBP followed by a greater decline after the age of 78 than those who did not [25]. A longitudinal study from the Kungsholmen Project revealed that individuals who developed dementia showed a greater decline in BP, especially in SBP, compared with those who remained free of dementia [26, 27]. In addition, the Adult Changes in Thought Study (*n*=2,356) reported that the mean SBP of subjects aged 64-75 who developed dementia was consistently higher and declined more sharply during the two years before diagnoses compared with those who did not [29]. However, some of the changes in BP trajectory may be caused by suffering from or progressing to dementia, which weakens their predictive significance for the development of dementia, and these changes are not formed naturally in the general population. From aforementioned studies, it is infeasible to identify meaningful subgroups of BP trajectory as well as their respective characteristics.

Moreover, the effects of BP change trajectory on the incidence of dementia in certain specific populations, such as the oldest-old (≥ 80 years old), women or men, and patients with hypertension, have not been well established. Previous studies have reported an age-varying association between mean BP and risk of dementia [29, 30]. It was found that the mean SBP was higher in subjects who developed dementia at an age lower than 75, but not in those who developed dementia after the age of 75 [29]. A lower BP was associated with a higher risk of dementia in individuals aged over 75 [30]. An earlier study suggested that there were gender differences in BP trajectory throughout the life, which might result in an inconsistent association between BP trajectory and dementia for men and women [24]. Apart from age and gender, the diagnosis of hypertension is also related to antihypertensive treatment and stable high BP levels. Existing studies have shown that antihypertensive drugs are associated with a decreased risk of dementia [31, 32]. Since high BP levels are deemed to affect the development of dementia [3], this factor needs to be well controlled. According to the general practice, only two repeated BP values are measured within one day, which may lead to errors due to various reasons. Therefore, the diagnosis of hypertension may be a better indicator for a stable BP level than the two BP measurements.

In the present study, we aimed to investigate the association between the trajectory of BP change and the risk of dementia and to explore the differences in age, gender, and hypertension subgroups using a nationally representative sample of adults aged ≥60 years. We hypothesized that BP trajectory patterns would have predictive significance for subsequent dementia events and there were differences in age, gender, and hypertension subgroups.

## RESULTS

### Basic characteristics of the study population

Of the 10,660 participants, 64.5% were aged 80-115 at the first visit, with female subjects accounting for 54.0% (Table 1). After a median follow-up of 5.9 years (the interval between the third and the first visit, interquartile range 4.8-6.3), the incidence rate of dementia was 9.8%.

**Table 1:** Demographic and clinical characteristics of CLHLS participants by SBP trajectory classes.

**Characteristics**	**Overall** **(*n*=10660)**	**Class 1** **(*n*=9219)**	**Class 2** **(*n*=639)**	**Class 3** **(*n*=586)**	**Class 4** **(*n*=216)**	***P* value**
80-115 years old ^a^	6881(64.5)	5908(64.1)	500(78.2) ^b^	341(58.2) ^b^	132(61.1)	<0.001
Female	5755(54.0)	4898(53.1)	390(61.0) ^b^	340(58.0)	127(58.8)	<0.001
Han nationality	9916(93.0)	8583(93.1)	604(94.5)	534(91.1)	195(90.3)	0.045
Education						
No schooling	6008(56.4)	5149(55.9)	389(60.9)	344(58.7)	126(58.3)	0.125
Primary school	3439(32.3)	2997(32.5)	193(30.2)	179(30.5)	70(32.4)	
White-collar	964(9.0)	850(9.2)	59(9.2)	34(5.8) ^b^	21(9.7)	0.046
Average household income (yuan)						
< 5000	5020(47.1)	4333(47.0)	333(52.1)	265(45.2)	89(41.2)	0.013
5000-19999	3955(37.1)	3400(36.9)	226(35.4)	232(39.6)	97(44.9)	
Place of residence						
City	2294(21.5)	2034(22.1)	135(21.1)	83(14.2) ^b^	42(19.4)	0.001
Town	3353(31.5)	2894(31.4)	195(30.5)	203(34.6)	61(28.2)	
Smoking status						
Current	1695(15.9)	1500(16.3)	79(12.4)	86(14.7)	30(13.9)	0.198
Past	2076(19.5)	1791(19.4)	131(20.5)	114(19.5)	40(18.5)	
Alcohol use						
Current	1715(16.1)	1499(16.3)	90(14.1)	100(17.1)	26(12.0)	0.325
Past	1762(16.5)	1534(16.6)	108(16.9)	87(14.8)	33(15.3)	
Regular exercise						
Current	3068(28.8)	2704(29.3)	144(22.5)	162(27.6)	58(26.9)	<0.001
Past	1811(17.0)	1572(17.1)	129(20.2)	83(14.2)	27(12.5)	
Sleep quality						
Very good or good	6240(58.5)	5386(58.4)	368(57.6)	371(63.3)	115(53.2)	0.002
Fair	2987(28.0)	2620(28.4)	180(28.2)	131(22.4) ^b^	56(25.9)	
Sleep duration (hours)	8.00(4.00)	8.00(4.00)	8.00(4.00)	8.00(4.00)	8.00(3.00)	0.196
Living alone	1621(15.2)	1413(15.3)	98(15.3)	82(14.0)	28(13.0)	0.654
Heart rate (beat/minute)	73(12)	73(12)	73(12)	74(13)	73(13)	0.417
Body mass index (kg/m^2^)	20.05(5.31)	20.00(5.23)	20.24(5.34)	20.57(5.19) ^**^	21.38(5.89) ^**^	<0.001
Diabetes	1659(15.6)	1437(15.6)	114(17.8)	75(12.8)	33(15.3)	0.114
Heart disease	2272(21.3)	1935(21.0)	158(24.7)	121(20.6)	58(26.9)	0.028
Cerebrovascular disease	1800(16.9)	1535(16.7)	126(19.7)	92(15.7)	47(21.8)	0.041
Respiratory disease	2076(19.5)	1812(19.7)	134(21.0)	83(14.2) ^b^	47(21.8)	0.006
Cancer	900(8.4)	783(8.5)	65(10.2)	36(6.1)	16(7.4)	0.078
Dementia	1049(9.8)	915(9.9)	75(11.7)	40(6.8)	19(8.8)	0.030

SBP, systolic blood pressure. Data are obtained at the third visit unless noted and expressed as numbers (percentages) or median (interquartile range). Class 1, normal SBP; class 2, stabilized SBP; class 3, elevated SBP; and class 4, persistently high SBP.

^a^Obtained at the first visit.

^b^There are statistically significant differences in the pairwise comparison between this class and class 1 trajectory.

### Latent growth mixture modeling

The fit indices for two- to six-class LGMM for SBP are presented in Supplementary Table 1. The values of loglikelihood, AIC, BIC, and SSA-BIC were found to continuously decline as the class increased. The LMR-test suggested that the four-class model outperformed the three-class model (*p* = 0.0019) while the five-class model outperformed the four-class model (*p* = 0.0307). However, the value of entropy indicated a higher classification accuracy of the four-class model relative to the five-class model. Besides, it appeared unreasonable that the smallest class of the five-class model contained only 0.85% of the total sample. Therefore, the four-class model was identified as the optimal fitting model to represent the changes of SBP trajectory. Similarly, based on a comprehensive comparison over fit indices, the three-class and four-class model were identified as the optimal fitting model to represent the changes of DBP and PP trajectory respectively (Supplementary Tables 2, 3).

### Characteristics of BP trajectory subgroups

**Figure 1:**
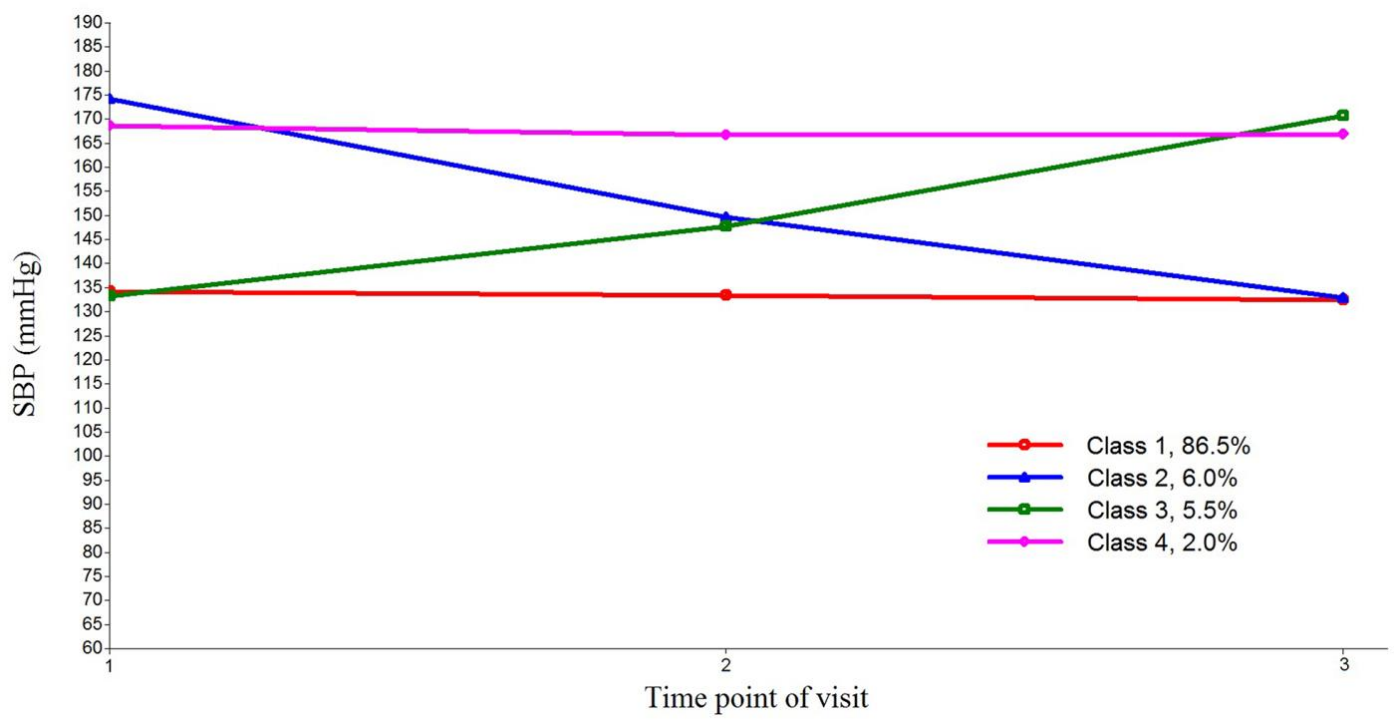
**Four latent trajectories of systolic blood pressure (SBP) for Chinese older people.** The latent growth mixture model was used to estimate the SBP trajectories. Class 1, normal SBP; class 2, stabilized SBP; class 3, elevated SBP; and class 4, persistently high SBP.

The trends and changes of four-class SBP trajectory, three-class DBP trajectory, and four-class PP trajectory are shown in Figure 1 and Supplementary Figures 1, 2 respectively. During the three follow-up visits, 86.5% of the participants maintained a normal SBP at about 135 mmHg and were classified into class 1 trajectory of SBP. Class 2 trajectory included 6.0% of the participants with the stabilized SBP declining from 175 to 135 mmHg. Class 3 trajectory included approximately 5.5% of the participants with an elevated SBP rising from 135 to 170 mmHg. Class 4 trajectory included 2.0% of the participants who had a persistently high SBP at about 170 mmHg. For DBP, 96.8% of the participants maintained a normal DBP at about 80 mmHg; 1.9% had their stabilized DBP declining from 120 to 80 mmHg; and only 1.2% had an elevated DBP rising from 80 to 120 mmHg. The participants who maintained a normal PP at 50 mmHg and a persistently high PP at 85 mmHg accounted for 83.4% and 1.7% of the total sample, respectively. Meanwhile, 8.5% and 6.4% of the participants had a stabilized or elevated PP that fluctuated between 50 and 85 mmHg, respectively.

Compared with the subjects maintaining a normal SBP, those having a stabilized SBP were more likely to be “80-115 years old” and “female”. Moreover, the subjects having an elevated SBP were more likely to be “60-79 years old”, “engaged in other works”, “not a city resident”, “having a higher body mass index”, and “reporting no respiratory disease”, and the subjects having a persistently high SBP were more likely to “have a higher body mass index”. In the population with missing values, similar characteristics as above were detected (Supplementary Table 4). Compared with the subjects maintaining a normal DBP (Supplementary Table 5), those having an elevated DBP were more likely to be “80-115 years old”. Compared with the subjects maintaining a normal PP (Supplementary Table 6), those having a stabilized PP were more likely to be “80-115 years old”, “female”, “illiterate”, “not in exercise currently”, and “suffering from heart disease”, and those having an elevated PP were more likely to be “rich”, “not a city resident”, “having a higher body mass index”, and “reporting no respiratory disease and dementia”. Moreover, the subjects having a persistently high SBP were more likely to be “female”, “non-rural resident”, and “having a higher body mass index”.

### Effects of SBP trajectory on the risk of dementia

**Figure 2:**
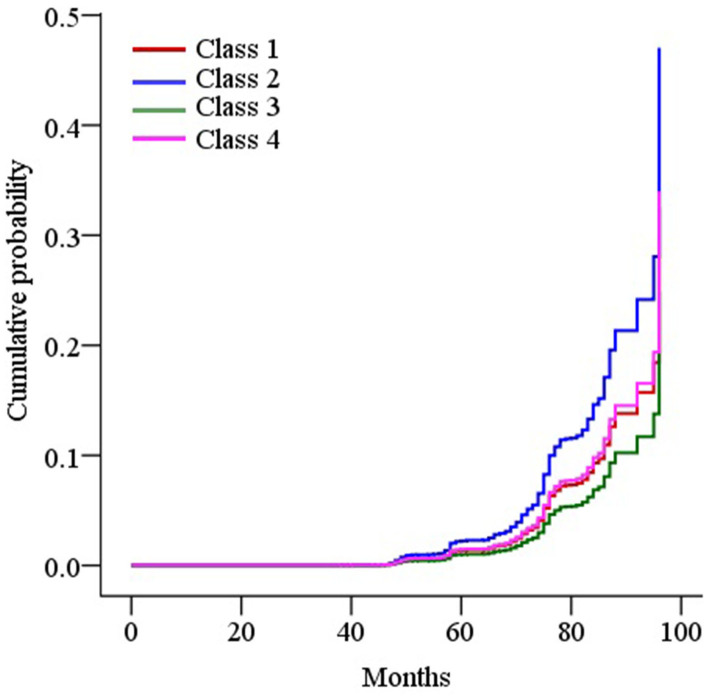
**Survival curves of the cumulative incidence of dementia by trajectory classes of systolic blood pressure (SBP) in the final adjusted model.** The Cox-proportional hazard model was used to plot the survival curves. Class 1, normal SBP; class 2, stabilized SBP; class 3, elevated SBP; and class 4, persistently high SBP.

According to primary analyses of the final adjusted model (Table 2), the subjects having a stabilized SBP involved a higher risk of dementia compared with those having a normal SBP [adjusted HR (aHR): 1.62; 95% CI: 1.27-2.07] or an elevated SBP (aHR: 2.22; 95% CI: 1.51-3.28). Figure 2 demonstrates the adjusted cumulative incidence of dementia by SBP trajectory.

**Table 2:** Effects of SBP trajectory on the risk of dementia.

**Variables**	**Model 1**	**Model 2**	**Model 3**	**Model 4**
**Normal SBP as reference**				
Stabilized SBP	1.74(1.38, 2.20) ***	1.75(1.38, 2.22) ***	1.79(1.41, 2.27) ***	1.62(1.27, 2.07) ***
Elevated SBP	0.64(0.46, 0.88) **	0.70(0.51, 0.96) *	0.70(0.51, 0.96) *	0.73(0.53, 1.00)
Persistently high SBP	0.96(0.61, 1.51)	1.03(0.65, 1.62)	1.03(0.65, 1.62)	1.06(0.66, 1.69)
**Persistently high SBP as reference**				
Stabilized SBP	1.82(1.10, 3.00) *	1.71(1.03, 2.83) *	1.74(1.05, 2.88) *	1.53(0.91, 2.56)
Elevated SBP	0.67(0.39, 1.15)	0.68(0.39, 1.18)	0.68(0.39, 1.17)	0.69(0.40, 1.20)
**Elevated SBP as reference**				
Normal SBP	1.57(1.14, 2.15) **	1.43(1.04, 1.97) *	1.44(1.05, 1.97) *	1.38(1.00, 1.90)
Stabilized SBP	2.73(1.86, 4.01) ***	2.51(1.71, 3.69) ***	2.57(1.75, 3.77) ***	2.22(1.51, 3.28) ***

SBP, systolic blood pressure. Hazard ratios (95% confidence intervals) are presented. Model 1 was adjusted for no covariates. Model 2 was adjusted for age, gender, ethnic group, education, primary occupation before retirement, average household income, and place of residence. Model 3 was adjusted for model 2 plus smoking, alcohol use, regular exercise, sleep quality, sleep duration, and living alone. Model 4 was adjusted for model 3 plus heart rate, body mass index, hypertension, diabetes, heart disease, cerebrovascular disease, respiratory disease, and cancer. * *P* <0.05, **P < 0.01, *** *P* < 0.001.

The aforementioned effects were observed with a stronger significance of aHR 1.87 (95% CI: 1.44-2.43) and 2.67 (95% CI: 1.69-4.26) in the subgroup aged 80-115 years at the first visit (Supplementary Table 7), but not in the subgroup aged 60-79 years. In terms of gender difference, we observed stronger effects in female subjects with the aHR of 1.98 (95% CI: 1.45-2.70) and 3.21 (95% CI: 1.93-5.34), but not in male subjects. In the subjects suffering from hypertension at the first visit, only those having a stabilized SBP appeared to involve a higher risk of dementia compared with those having a normal SBP (aHR: 2.94; 95% CI: 2.03-4.27). However, in the subjects not suffering from hypertension at the first visit, those having a stabilized SBP (aHR: 3.60; 95% CI: 2.12-6.12) or normal SBP (aHR: 1.90; 95% CI: 1.29-2.80) also appeared to involve a higher risk of dementia compared with those having elevated SBP.

In all the sensitivity analyses (Supplementary Table 8), we observed similar effects to those in primary analyses. Additionally, it was found that the subjects having a normal SBP involved a higher risk of dementia compared with those having an elevated SBP after excluding subjects with a history of heart disease or screened as moderate or severe cognitive impairment at the first visit.

### Effects of DBP trajectory on the risk of dementia

According to the primary and sensitivity analyses of the final adjusted model, no statistically significant association was observed between the DBP trajectory and the risk of dementia (Supplementary Tables 9, 10). Supplementary Figure 3 shows the adjusted cumulative incidence of dementia by DBP trajectory.

In the subjects suffering from hypertension at the first visit (Supplementary Table 11), the subjects having an elevated DBP appeared to involve a higher risk of dementia compared with those having normal DBP (aHR: 4.37; 95% CI: 1.96-9.73). However, no statistically significant association was observed in other subgroups.

### Effects of PP trajectory on the risk of dementia

According to primary analyses of the final adjusted model (Supplementary Table 12), the subjects having a stabilized PP appeared to involve a higher risk of dementia compared with those having a normal PP (aHR: 1.52; 95% CI: 1.24-1.88) or elevated PP (aHR: 2.12; 95% CI: 1.48-3.04). Besides, the subjects having a normal PP involved a higher risk of dementia compared with those having an elevated PP (aHR: 1.39; 95% CI: 1.02, 1.90). Supplementary Figure 4 shows the adjusted cumulative incidence of dementia by PP trajectory.

The aforementioned effects were observed with a stronger significance in the subgroup aged 80-115 years at the first visit and in female subjects (Supplementary Table 13), but were not observed in the subgroup aged 60-79 years. In the male subjects, the subjects having a stabilized PP appeared to involve a higher risk of dementia compared with those having a normal PP (aHR: 1.45; 95% CI: 1.04-2.02). In the subjects suffering from hypertension at the first visit, those having a stabilized PP appeared to involve a higher risk of dementia compared with those having a normal SBP (aHR: 3.00; 95% CI: 2.12-4.24), persistently high PP (aHR: 3.06; 95% CI: 1.41-6.66), or elevated PP (aHR: 3.11; 95% CI: 1.69-5.72). In the subjects not suffering from hypertension at the first visit, those having a normal PP (aHR: 1.61; 95% CI: 1.10-2.37) or stabilized PP (aHR: 2.13; 95% CI: 1.34, 3.38) appeared to involve a higher risk of dementia compared with those having an elevated PP.

After excluding subjects with a history of heart disease or screened as moderate or severe cognitive impairment at the first visit, we observed similar effects to those in primary analyses (Supplementary Table 14). However, after excluding subjects with a history of diabetes or diagnosed of cerebrovascular disease at the first visit, the subjects having a normal PP no longer appeared to involve a higher risk of dementia compared with those having an elevated SBP in the final adjusted model.

## DISCUSSION

In this nationwide cohort study in China, stabilized SBP was found to be associated with a higher risk of dementia compared with normal SBP and elevated SBP in the total sample, but not in the subgroups of the oldest-old, women, and subjects without hypertension at baseline. Similarly, stabilized PP was associated with a higher risk of dementia compared with normal PP and elevated PP in the total sample, but not in the subgroups of the oldest-old, women, and subjects with hypertension at baseline. However, there was no strong evidence for the direct association between DBP trajectory and dementia.

In this study, we identified four unique BP/PP trajectories: normal, stabilized, elevated, and persistently high BP/PP. The proportion of normal BP in the present study was higher than that in prior studies [17, 18], probably because our subjects were enrolled from longevity areas and had fewer harmful BP changes than the general population. Consistent with our study, the Cardiovascular Health Study reported similar normal, stabilized, and elevated SBP trajectories among 4,067 subjects with a median age of 77 years [17]. The differences in age, gender, body mass index, and risk of cardiovascular and cerebrovascular disease among SBP trajectory groups were also observed as consistent as in our study [17]. Our findings suggest that the occurrence of stabilized SBP should not be considered as a complication of heart disease, diabetes, and cerebrovascular diseases, but higher body mass index resulted in an elevated and persistently high PP. The mechanisms by which these abnormal trajectories are generated need to be further studied.

Consistent with our findings, previous studies also demonstrated that SBP tended to decline a few years before the onset of dementia [24–26, 33, 34]. Besides, the SBP remained consistently at a level above the normal value before it began to decline [24–26, 33]. It was repeatedly reported that individuals with a higher SBP in midlife involved a significantly increased risk of dementia in their later life [3, 4]. Several decades-long follow-up studies demonstrated that the SBP of those subjects who developed dementia tended to increase more significantly from midlife to late-life and thereafter declined more in the years before the dementia onset [24, 25, 35]. An earlier study suggested that the trajectory of change, rather than the current BP, might be most useful in identifying a subsequent diagnosis of dementia [36], but the causal association between SBP trajectory and neurodegeneration remained unclear since dementia had a decades-long prodrome. The pathology of Alzheimer’s disease may have been observed as early as 20 years ahead of diagnosis [37]. It is undeniable that the progression to dementia may cause a decline in SBP, which needs to be confirmed by pathological studies in the prodromal stage of dementia. However, stabilized SBP still has predictive significance for the occurrence of dementia. Inconsistent with previous studies, the present research did not find any detrimental effect of persistently high or elevated SBP on dementia [29, 33]. In contrast, we found that elevated SBP was associated with a reduced risk of dementia in subjects without hypertension, heart disease, or cognitive impairment, compared with those having a normal SBP. This finding conflicted with the accumulated evidence that BP variation was positively associated with the risk of dementia regardless of the direction [8–11]. In a practical sense, SBP should not be elevated by intervention for the purpose to reduce the risk of dementia. Repeated studies are required to confirm this finding.

One mechanism may exist that mid-life hypertension and late-life hypotension independently affect dementia. Long-term high BP starting from midlife can lead to a series of cerebrovascular diseases, such as white matter damage, asymptomatic cerebral infarction, and clinical stroke. The ischemic brain injuries caused by these diseases may act alone or in combination with neurodegenerative changes in late life to promote the clinical manifestations of dementia syndrome [38]. Higher blood pressure would result in more severe cerebral atherosclerosis, which is also related to cerebral neurodegenerative diseases and the clinical manifestations of dementia [39, 40]. There may be two pathways linking low BP with dementia. First, neuroimaging studies reported that low BP was associated with more severe white matter lesions and atrophy of the hippocampus [41, 42], which significantly increased the risk of dementia [43, 44]. Second, intermittent or persistent hypotension may further damage cerebral blood perfusion and lead to more extensive cerebral ischemia, which accelerates the clinical manifestation of dementia syndrome [26]. Another mechanism may be attributed to the combined effects of mid-life hypertension and late-life hypotension on dementia. When the systemic BP is low, the impairment of brain autoregulation caused by chronic hypertension will lead to a decreased ability in maintaining stable blood flow, especially prone to the decrease of cerebral blood flow [33, 45]. The latter has been associated with pathogenic brain changes [33, 46].

This study found that, in the group of hypertension at entry, elevated DBP was associated with a higher risk of dementia than normal DBP. Inconsistent with our finding, previous studies reported an association between declined DBP and a higher risk of dementia. A recent meta-analysis supported that midlife high DBP was associated with an increased risk of dementia, while late-life low DBP was associated with an increased risk of dementia [47], which suggests that there may be a certain declining pattern of DBP affecting the risk of dementia. The Kungsholmen analyses and the Honolulu-Asia Aging Study reported that individuals who developed dementia showed a decline in DBP during the 3-year and 6-year period before diagnosis, respectively [25, 26]. Probably because only a few participants are classified into the elevated DBP trajectory group, unusually large statistical effects were detected in this study, suggesting a certain possibility of errors. Consistent with our results, the Gerontological Regional Database study observed that incident dementia cases exhibited a greater decline in PP over 5 years among the very old [48]. The mechanisms underlying the association between the decline in PP and an increased dementia risk remain unclear. A decline in PP may indicate a decrease in blood ejection and stroke volume, which can be associated with dementia through impaired cerebral blood flow [49]. Besides, the decline in PP may also be associated with cerebral vascular lesions and dysregulation of BP caused by severe cerebral atherosclerosis, which may lead to dementia [49].

In subgroup analyses, the association between stabilized SBP/PP and dementia was observed only in subjects aged ≥ 80 years but not in those aged 60-79 years. According to the existing evidence, there have been mixed results regarding the relationship between a declined SBP and dementia in the same age group. A decades-long cohort study showed that the pattern of midlife hypertension and late-life hypotension was associated with incident dementia only in the younger group [33]. The Adult Changes in Thought Study found that SBP declined more significantly in those subjects who developed dementia at a younger age, but not in those who developed dementia at an older age [29]. Consistent with our findings, the Gerontological Regional Database study reported that individuals who developed dementia exhibited a greater SBP decline in the very old subjects [48]. This may be partially explained by the age differences in the incidence of dementia. It has been well established that the incidence rate of dementia is much higher in the very old than in the young old. These findings suggest that age is likely to be an important factor for consideration when estimating the risk of BP decline on dementia.

Few studies have thus far specifically examined the gender differences in the association between SBP trajectory and dementia. Out of our expectation, statistically significant results were only observed in older women but not in older men. A 37-year cohort study confirmed that older women who developed dementia had a steeper decline in SBP during the five years before diagnosis [24]. In contrast, a 30-year cohort study showed that older men with a decreased SBP had poor cognitive performance [50]. In another 32-year prospective study, older men who developed dementia showed a greater SBP decline during the six years before diagnosis [25]. This is possibly attributed to the difference in the SBP trajectory composition between female and male groups in our study: there were more subjects with stabilized SBP in the female group than in the male group. With more subjects being classified as stabilized SBP, it was more likely to observe statistically significant results in the female group.

In our study, stabilized SBP was also found to increase the risk of dementia in subjects without hypertension, indicating that the trajectory of SBP change could affect dementia in a way independent of late-life hypertension. Besides, the effect in the hypertension group was stronger than that in the non-hypertension group, suggesting that late-life hypertension can also affect the relationship between the trajectory of SBP change and dementia. Consistent with our study, an association between a subsequent steep decline in SBP and an increased risk of dementia was reported in individuals without hypertension and/or not receiving antihypertensive therapy at midlife [34]. In subjects who developed dementia, a steeper decline in SBP during the five years before diagnosis was observed in individuals receiving antihypertensive treatment than in individuals never receiving antihypertensive treatment [24]. However, the Honolulu-Asia Aging Study reported no association between SBP trajectory and dementia in subjects who were currently on antihypertensive treatment [25], probably because this study only examined male subjects. In addition, stabilized PP was found to increase the risk of dementia in comparison with normal and persistently high PP only in the hypertension group, suggesting that late-life hypertension could affect the association between PP trajectory and dementia. In the non-hypertensive group, normal PP was found to increase the risk of dementia in comparison with elevated PP, which requires more comprehensive research to confirm.

One of the strengths of our study lies in the use of a large and nationally representative sample, which equips our results with a higher statistical power. The larger sample also provides the possibility of subgroup analyses. Second, our sensitivity analyses showed similar results when subjects involving a high risk of dementia were excluded, providing further evidence on the robustness of our findings. Third, to the best of our knowledge, this is the first study that explored the relationship between the trajectory of PP change and subsequent dementia. Fourth, this study determined the BP trajectory patterns up to 6-year follow-up among older adults free of dementia at entry. Compared with the previous researches in which BP trajectories were grouped based on whether dementia occurred or not, our study provided stronger evidence for the association between the decline in SBP from hypertension and the subsequent dementia.

Nevertheless, potential limitations should be acknowledged as well. First, since the median length of follow-up was only 6 years in this study, we could not confirm whether stabilized SBP/PP acted as a consequence of neurodegeneration or a risk factor for later dementia. Secord, because of data unavailability, this study was unable to detect any associations between BP trajectory and different types and degrees of dementia. Previous studies have found that certain specific BP characteristics had a unique effect on the vascular- and Alzheimer-specific pathology [51]. Individuals with the most advanced dementia were found to have the lowest BP [25]. Third, this study only measured the BP trajectory at three-time points. If more time points were covered, the measurements of BP trajectory would be more accurate. Fourth, dementia was assessed based on self- or proxy-reported hospital diagnosis, but it had not been confirmed by a doctor’s examination. Fifth, as a survival sample, the survival bias might affect some of our findings. Both hypertension and dementia can influence the risk of mortality, and thus people with baseline hypertension might have been excluded as participants due to death. Therefore, this study might underestimate the association between BP trajectory and dementia. Sixth, most of the measurements were reported by participants or their proxies, so the possibility of underreporting or misreporting might exist due to recall bias. Seventh, measurement bias might occur due to possible incorrect and missed diagnoses of dementia, as well as the lack of BP monitoring data. Finally, the effect of specific antihypertensive drugs on the risk of dementia could not be adjusted due to data insufficiency.

In the present nationwide cohort study, stabilized SBP was found to be associated with a higher risk of dementia only in the groups of the oldest-old, women, and subjects without hypertension at baseline, in comparison with normal SBP and elevated SBP. It was also found that stabilized PP was associated with a higher risk of dementia only in the groups of the oldest-old, women, and subjects with hypertension at baseline, in comparison with normal PP and elevated PP. These findings suggest that stabilized SBP and PP in late life have predictive significance for the occurrence of dementia. Age, gender, and late-life hypertension should be comprehensively considered when estimating the risk of BP decline on dementia. Further follow-ups from middle to late life are required to reveal the associations between the whole process of BP trajectory and dementia.

## MATERIALS AND METHODS

### Study population

The Chinese Longitudinal Healthy Longevity Survey (CLHLS) is an ongoing and prospective cohort study in China to investigate the determinants of health and longevity of older adults. A detailed description of the study design can be found elsewhere [52–54]. The CLHLS was carried out every 2-4 years from 1998 to 2018. Half of the counties and cities in 23 provinces/municipalities/autonomous regions in China were selected as its study sites. For the subjects who were dead or lost to follow-up, new participants would be enrolled according to the same gender and age nearby. The surveys were conducted in the participants’ residential places by well-trained interviewers with structured questionnaires. Family members, caregivers, or institutional staff as proxy respondents were interviewed when the participants were unable to answer questions by themselves. The current study was based on 8 waves of the CLHLS from 1998 to 2018. From a total of 101,779 individuals, we included 21,783 subjects who were followed up for three times in the period of 1998-2018. Then we excluded 6 subjects who were aged <60 years at the first visit, 2038 subjects who were diagnosed of dementia at the first and second visits, 1089 subjects who missed BP measurements at three visits, 752 subjects who missed assessments of dementia at the third visit, and 7238 subjects who were followed up repeatedly. Eventually, the entire study sample consisted of 10,660 subjects (Supplementary Figure 5).

The CLHLS study was approved by the Biomedical Ethics Committee of Peking University, Beijing, China (IRB00001052-13074), and informed consent was obtained from all participants or their proxy respondents.

### Assessment of dementia

Dementia at each visit was assessed based on the self- or proxy-reported hospital diagnosis (“have you been diagnosed with dementia by the hospital?”). Only subjects who responded ‘yes’ to the question were defined as incident dementia [55]. The diagnosis of dementia at the third visit was the outcome variable for this analysis.

### Measurements of BP

After at least 5 minutes of rest, interviewers would test the BP measurement twice for each participant with a mercury sphygmomanometer (upper arm type; Yuyue, Jiangsu, China) on the right arm at the same height as the heart, and the interval between the two measurements should be at least one minute. For bedridden participants, BP was tested in the recumbent position. Korotkoff phase I and phase V were referred to as the SBP and DBP value respectively. In subsequent analyses, the average value of SBP and DBP was calculated from two measurements. PP was obtained from the difference between SBP and DBP [56].

### Measurements of covariates

Sociodemographic characteristics, lifestyle and health behaviors, and medical examination results were considered as potential confounders by referencing to the existing literature [24, 25, 33, 34]. The sociodemographic characteristics included age (60-79/80-115 years old), gender (female/male), ethnic group (Han/minority), education (no schooling/primary school/high school and above), primary occupation before retirement (white-collar/others), average household income (< 5000/5000-19999/≥ 20000 yuan), and place of residence (city/town/rural areas). The lifestyle and health behaviors included smoking status (current/past/never), alcohol use (current/past/never), regular exercise (current/past/never), sleep quality (very good or good/fair/bad or very bad), sleep duration (hours), and living alone (yes/no). The medical examination results included heart rate (beat/minute), body mass index (kg/m^2^), hypertension (yes/no), diabetes (yes/no), heart disease (yes/no), cerebrovascular disease (yes/no), respiratory disease (yes/no), and cancer (yes/no). All variables were obtained at the third visit, except for age (at the first visit). The heart rate referred to the number of heartbeats per minute measured by the interviewer with a stethoscope. Hypertension, diabetes, heart disease, cerebrovascular disease, respiratory disease, and cancer were assessed based on the self- or proxy-reported hospital diagnosis (“have you been diagnosed with those diseases by the hospital?”). Only participants who responded ‘yes’ to the questions were defined as incident events.

### Statistical analysis

Continuous variables were presented as median (interquartile range) in view of that they are all non-normally distributed by normality tests. Categorical variables were presented as numbers (percentages). The differences in demographic and clinical characteristics between BP trajectory classes were compared using the chi-square test for continuous variables and the Kruskal-Wallis *H* test for categorical variables.

The latent growth mixture model (LGMM) was used to estimate the BP trajectory across the three visits. LGMM was a method for identifying multiple unobserved subpopulations with varying intercepts and slopes, describing the longitudinal change of each subpopulation [57], and examining the differences in change among latent subpopulations [58]. The trajectory of change in BP across time was modeled with two latent variables: one was the latent intercept growth factor that reflects the initial level of the BP, and the other one was the latent slope growth factor that represents the rate of BP change. The categorical latent variables (classes) in LGMM were used to model different subpopulations.

The latent classes of BP trajectory were identified in two steps. First, we assumed three change functions (linear, quadratic, and freely estimated) to determine the best way to model the trajectory change over time. Given data of only three visits, the BP trajectory was modeled by the specified linear change. Second, to identify the appropriate number of classes for the most desirable fit, we established two- to six-class unconditional LGMM models (with no covariates or predictors). A variety of model fitting indices were used to evaluate the goodness of LGMM. Lower values on loglikelihood, Akaike Information Criterion (AIC), Bayesian Information Criterion (BIC), and sample-size adjusted BIC (SSA-BIC) indicated a better model fit. A higher value of entropy indicated a higher classification accuracy. The Lo-Mendell-Rubin adjusted likelihood ratio test (LMR-test) and bootstrap likelihood ratio test (BLRT) were used to compare the *k*-class model with (*k*-1)-class models. The significant *p*-value suggested that the *k*-class model was more suitable than the (*k*-1)-class model. Moreover, the size of the smallest class was required not to be less than 1.0% or 25 subjects [59].

The Cox-proportional hazard models were used to analyze the effects of BP trajectory on the risk of dementia and to plot the survival curve of the cumulative incidence of dementia by BP trajectory classes. Hazard ratio (HR) and its corresponding 95% confidence interval (CI) were used to quantify the extent of effects. We established four models in total. Model 1 was the basic model taking the interval between the third and the first visit as the survival time. The influence of confounders was further analyzed in three additional models. In model 2, we adjusted the sociodemographic characteristics of the subjects. In model 3, we added the variables related to lifestyle and health behaviors. In model 4, we further added the medical examination results. To further analyze the effects of BP trajectory on the risk of dementia in specific populations and to examine the heterogeneity, we stratified the sample into different subgroups and repeated the analyses above. We considered the factors of age, gender, and diagnosis of hypertension at the first visit. To test the robustness of the effects of BP trajectory on the risk of dementia, the following sensitivity analyses were performed: (1) excluding subjects with a history of heart disease at the first visit; (2) excluding subjects with a history of diabetes at the first visit; (3) excluding subjects with the diagnosis of cerebrovascular disease at the first visit; (4) excluding subjects screened as moderate or severe cognitive impairment at the first visit based on the Mini-Mental State Examination score (≤ 20).

Overall, there was a small percentage of missing data for all variables (3.28%), which was compensated by the multiple imputations method. In all the analyses, a two-sided *P* value < 0.05 was considered statistically significant. LGMM analyses were conducted in Mplus version 7.0 (Muthén and Muthén, Los Angeles, CA, USA). All other statistical analyses were performed in SPSS 22.0 software (IBM SPSS Inc., New York, NY, USA).

## Supplementary Material

Supplementary Figures

Supplementary Tables 1, 2, and 3

Supplementary Table 4

Supplementary Tables 5 and 6

Supplementary Table 7

Supplementary Tables 8 to 12

Supplementary Table 13

Supplementary Table 14
