# Supplementary material for: Trajectory patterns of blood pressure change up to six years and the risk of dementia: a nationwide cohort study
Source: Aging (Albany NY). 2021 Jul 1;13(13):17380–406. doi: 10.18632/aging.203228 (PMC8312414; doi:10.18632/aging.203228)
Supplement: Supplementary Figures [file aging-13-203228-s001.pdf]

SUPPLEMENTARY FIGURES

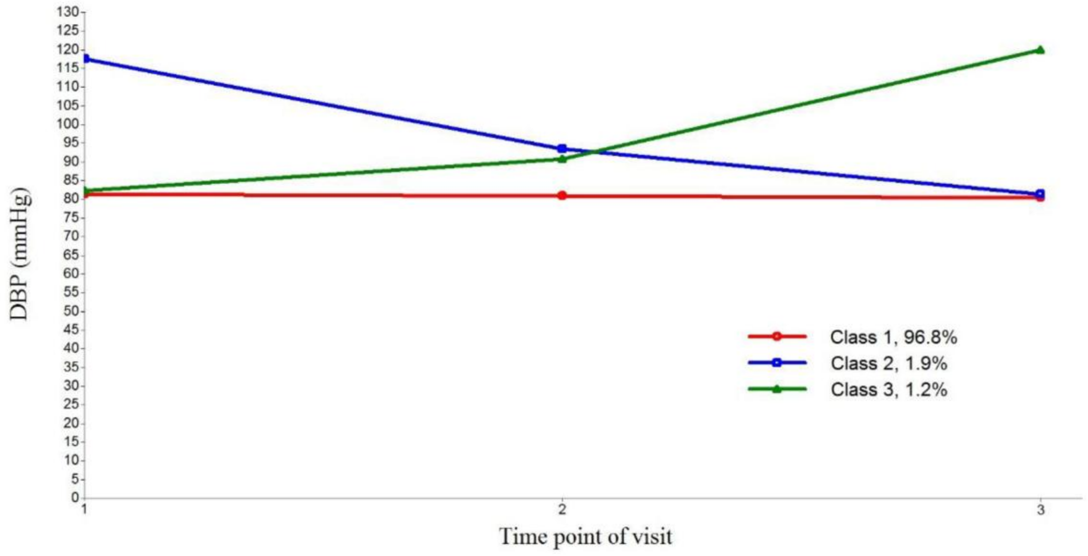

Supplementary Figure 1. Three latent trajectories of diastolic blood pressure (DBP) for Chinese older people. The latent growth mixture model was used to estimate the DBP trajectories. Class 1, normal DBP; class 2, stabilized DBP; class 3, elevated DBP.

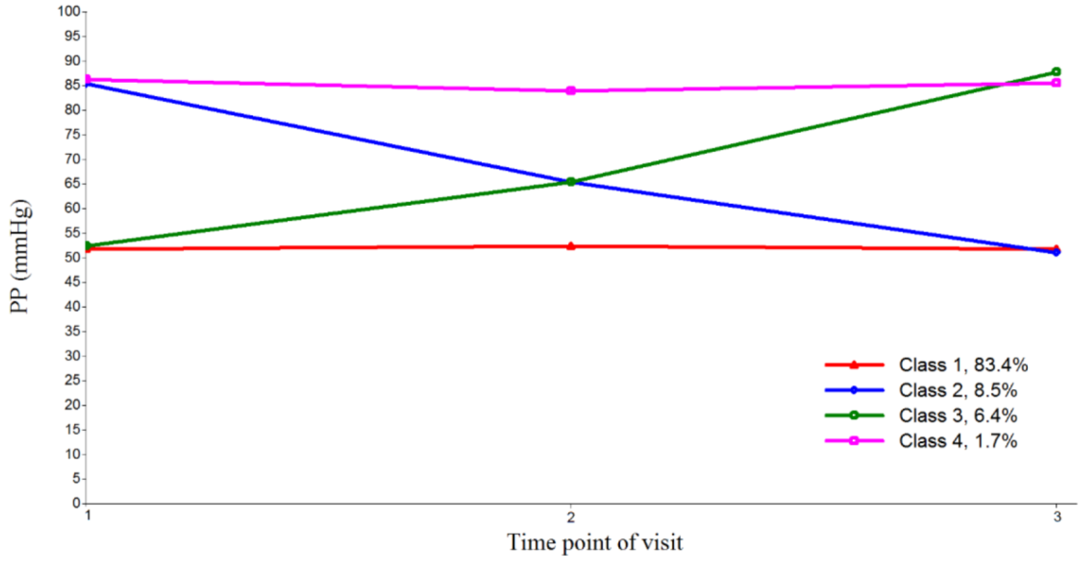

Supplementary Figure 2. Four latent trajectories of pulse pressure (PP) for Chinese older people. The latent growth mixture model was used to estimate the PP trajectories. Class 1, normal PP; class 2, stabilized PP; class 3, elevated PP; and class 4, persistently high PP.

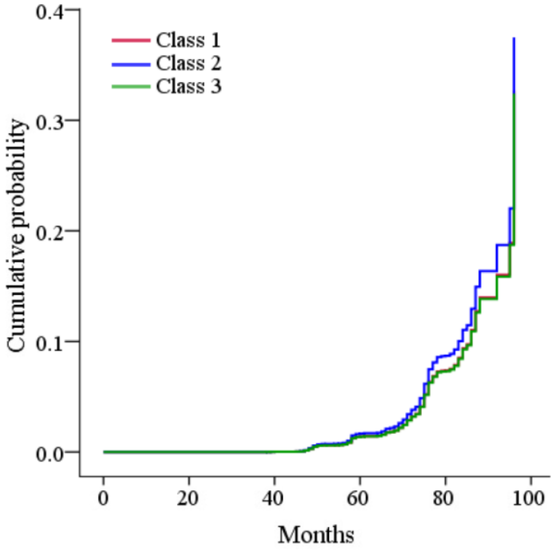

Supplementary Figure 3. Survival curves of the cumulative incidence of dementia by trajectory classes of diastolic blood pressure (DBP) in the final adjusted model. The Cox-proportional hazard model was used to plot the survival curves. Class 1, normal DBP; class 2, stabilized DBP; class 3, elevated DBP.

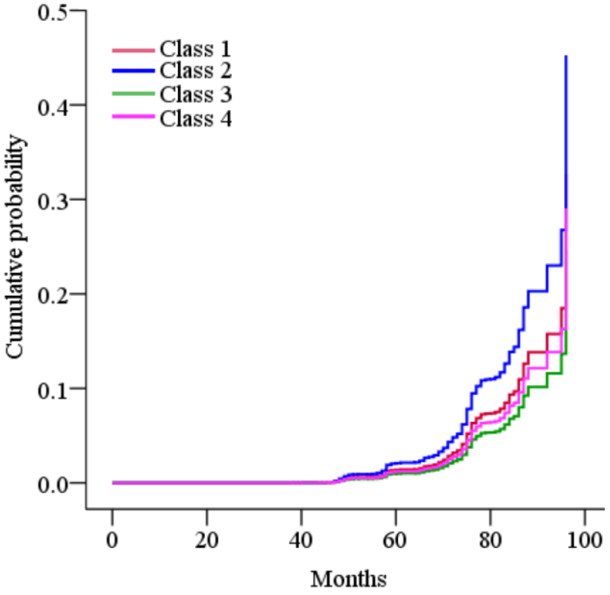

Supplementary Figure 4. Survival curves of the cumulative incidence of dementia by trajectory classes of pulse pressure (PP) in the final adjusted model. The Cox-proportional hazard model was used to plot the survival curves. Class 1, normal PP; class 2, stabilized PP; class 3, elevated PP; and class 4, persistently high PP.

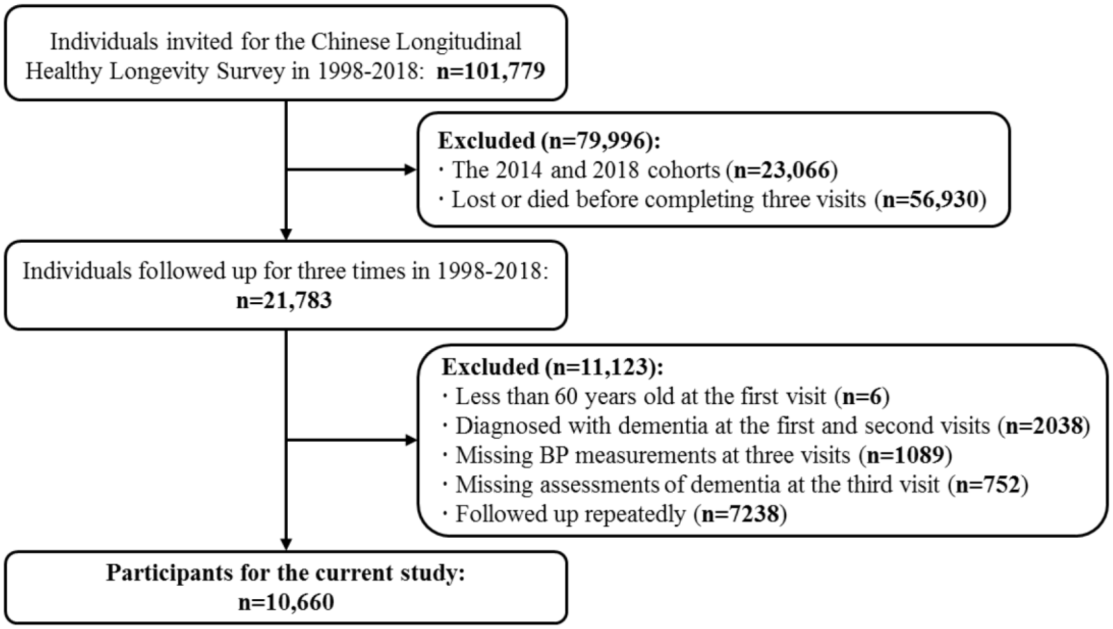

Supplementary Figure 5. Flowchart of the study population.
