# Supplementary material for: Trajectory patterns of blood pressure change up to six years and the risk of dementia: a nationwide cohort study
Source: Aging (Albany NY). 2021 Jul 1;13(13):17380–406. doi: 10.18632/aging.203228 (PMC8312414; doi:10.18632/aging.203228)
Supplement: Supplementary Tables 1, 2, and 3 [file aging-13-203228-s002.pdf]

Supplementary Table 1. Fit indices for two- to six-class growth mixture models for systolic blood pressure.

Classes	Loglikelihood	AIC	BIC	SSA-BIC	LMR-test	BLRT	Entropy	The size of the smallest class
2-class	-141295.934	282613.867	282693.884	282658.927	<0.0001	<0.0001	0.776	880(8.26%)
3-class	-141096.860	282221.720	282323.559	282279.069	<0.0001	<0.0001	0.770	633(5.94%)
**4-class**	**-141047.789**	**282129.579**	**282253.241**	**282199.218**	**0.0019**	**<0.0001**	**0.785**	**216(2.03%)**
5-class	-140998.462	282036.923	282182.408	282118.851	0.0307	<0.0001	0.748	91(0.85%)
6-class	-140987.180	282020.361	282181.669	282114.578	0.5608	<0.0001	0.758	38(0.36%)

AIC, Akaike information criterion; BIC, Bayesian information criterion; SSA-BIC, Sample-size adjusted BIC; LMR-test, Lo-Mendell-Rubin adjusted likelihood ratio test; BLRT, bootstrap likelihood ratio test.

Supplementary Table 2. Fit indices for two- to six-class growth mixture models for diastolic blood pressure.

Classes	Loglikelihood	AIC	BIC	SSA-BIC	LMR-test	BLRT	Entropy	The size of the smallest class
2-class	-124501.013	249024.026	249104.043	249069.087	< 0.001	< 0.001	0.957	211(1.98%)
**3-class**	**-124234.207**	**248496.414**	**248598.254**	**248553.763**	**< 0.001**	**< 0.001**	**0.957**	**133(1.25%)**
4-class	-124190.191	248414.382	248538.044	248484.021	0.079	< 0.001	0.932	74(0.69%)
5-class	-124165.559	248371.118	248516.603	248453.046	0.086	< 0.001	0.934	10(0.09%)
6-class	-124147.409	248340.819	248508.126	248435.035	0.003	< 0.001	0.940	10(0.09%)

AIC, Akaike information criterion; BIC, Bayesian information criterion; SSA-BIC, Sample-size adjusted BIC; LMR-test, Lo-Mendell-Rubin adjusted likelihood ratio test; BLRT, bootstrap likelihood ratio test.

Supplementary Table 3. Fit indices for two- to six-class growth mixture models for pulse pressure.

Classes	Loglikelihood	AIC	BIC	SSA-BIC	LMR-test	BLRT	Entropy	The size of the smallest class
2-class	-137086.193	274194.386	274274.403	274239.446	< 0.001	< 0.001	0.826	857(8.04%)
3-class	-136808.851	273645.702	273747.542	273703.052	< 0.001	< 0.001	0.793	722(6.77%)
**4-class**	**-136726.496**	**273486.992**	**273610.654**	**273556.631**	**0.008**	**< 0.001**	**0.796**	**184(1.73%)**
5-class	-136675.840	273391.679	273537.164	273473.607	0.270	< 0.001	0.759	131(1.23%)
6-class	-136646.442	273338.885	273506.193	273433.102	0.142	< 0.001	0.772	17(0.16%)

AIC, Akaike information criterion; BIC, Bayesian information criterion; SSA-BIC, Sample-size adjusted BIC; LMR-test, Lo-Mendell-Rubin adjusted likelihood ratio test; BLRT, bootstrap likelihood ratio test.
