# Supplementary material for: Trajectory patterns of blood pressure change up to six years and the risk of dementia: a nationwide cohort study
Source: Aging (Albany NY). 2021 Jul 1;13(13):17380–406. doi: 10.18632/aging.203228 (PMC8312414; doi:10.18632/aging.203228)
Supplement: Supplementary Table 4 [file aging-13-203228-s003.docx]

**Supplementary Table 4. Demographic and clinical characteristics with missing values of CLHLS participants by SBP trajectory classes.**

| **Characteristics** | **Overall** | **Class 1** | **Class 2** | **Class 3** | **Class 4** | ***P* value** | |  |
| --- | --- | --- | --- | --- | --- | --- | --- | --- |
| Ethnic group |  |  |  |  |  |  | |  |
| Han nationality | 9653(90.6) | 8374(93.1) | 592(94.7) | 503(90.8) | 184(91.1) | 0.043 | |  |
| Minority | 727(6.8) | 625(6.9) | 33(5.3) | 51(9.2) | 18(8.9) |  | |  |
| Missing | 280(2.6) |  |  |  |  |  | |  |
| Education |  |  |  |  |  |  | |  |
| No schooling | 5994(56.2) | 5136(55.9) | 388(61.0) | 344(58.8) | 126(58.3) | 0.118 | |  |
| Primary school | 3427(32.1) | 2988(32.5) | 191(30.0) | 178(30.4) | 70(32.4) |  | |  |
| High school and above | 1209(11.3) | 1069(11.6) | 57(9.0) | 63(10.8) | 20(9.3) |  | |  |
| Missing | 30(0.3) |  |  |  |  |  | |  |
| Primary occupation before retirement | |  |  |  |  |  | |  |
| White-collar | 964(9.0) | 850(9.2) | 59(9.2) | 34(5.8) ^a^ | 21(9.8) | 0.046 | |  |
| Others | 9681(90.8) | 8357(90.8) | 579(90.8) | 551(94.2) | 194(90.2) |  | |  |
| Missing | 15(0.1) |  |  |  |  |  | |  |
| Average household income (yuan) | |  |  |  |  |  | |  |
| < 5000 | 4732(44.4) | 4081(46.8) | 315(52.7) ^a^ | 252(45.7) | 84(42.4) | 0.021 | |  |
| 5000-19999 | 3736(35.0) | 3225(37.0) | 209(34.9) | 215(38.9) | 87(43.9) |  | |  |
| ≥ 20000 | 1596(15.0) | 1410(16.2) | 74(12.4) | 85(15.4) | 27(13.6) |  | |  |
| Missing | 596(5.6) |  |  |  |  |  | |  |
| Smoking status | |  |  |  |  |  | |  |
| Current | 1688(15.8) | 1494(16.3) | 79(12.4) | 85(14.6) | 30(13.9) | 0.207 | |  |
| Past | 2065(19.4) | 1781(19.4) | 130(20.4) | 114(19.6) | 40(18.5) |  | |  |
| Never | 6865(64.4) | 5908(64.3) | 428(67.2) | 383(65.8) | 146(67.6) |  | |  |
| Missing | 42(0.4) |  |  |  |  |  | |  |
| Alcohol use |  |  |  |  |  |  | |  |
| Current | 1702(16.0) | 1487(16.2) | 89(14.0) | 100(17.2) | 26(12.0) | 0.313 | |  |
| Past | 1758(16.5) | 1530(16.7) | 108(17.0) | 87(15.0) | 33(15.3) |  | |  |
| Never | 7141(67.0) | 6150(67.1) | 440(69.1) | 394(67.8) | 157(72.7) | |  | |
| Missing | 59(0.6) |  |  |  |  | |  | |
| Regular exercise |  |  |  |  |  | |  | |
| Current | 30545(28.6) | 2694(29.4) | 142(22.4) | 160(27.5) | 58(26.9) | | <0.001 | |
| Past | 1803(16.9) | 1564(17.0) | 129(20.3) | 83(14.3) | 27(12.5) | |  | |
| Never | 5750(53.9) | 4917(53.6) ^a^ | 363(57.3) | 339(58.2) | 131(60.6) | |  | |
| Missing | 53(0.5) |  |  |  |  | |  | |
| Sleep quality |  |  |  |  |  | |  | |
| Very good or good | 5168(48.5) | 4520(58.3) | 208(55.5) | 343(63.3) | 97(55.7) | | 0.002 | |
| Fair | 2481(23.3) | 2210(28.5) | 112(29.9) | 118(21.8) ^a^ | 44(23.6) | |  | |
| Bad or very bad | 1198(11.2) | 1026(13.2) | 55(14.7) | 81(14.9) | 36(20.7) ^a^ | |  | |
| Missing | 1813(17.0) |  |  |  |  | |  | |
| Sleep duration (hours) | 8.00(4.00) | 8.00(4.00) | 8.00(3.00) | 8.00(4.00) | 8.00(3.00) | | 0.392 | |
| Missing | 1816(17.0) |  |  |  |  | |  | |
| Living alone |  |  |  |  |  | |  | |
| Yes | 1609(15.1) | 1400(15.3) | 102(15.8) | 81(14.1) | 26(12.3) | | 0.536 | |
| No | 8955(84.0) | 7738(84.7) | 544(84.2) | 494(85.9) | 185(87.7) | |  | |
| Missing | 96(0.9) |  |  |  |  | |  | |
| Heart rate (beat/ minute) | 73(12) | 73(12) | 73(12) | 74(13) | 75(14) | | 0.402 | |
| Missing | 54(0.5) |  |  |  |  | |  | |
| Body mass index (kg/m^2^) | 20.03(5.31) | 20.00(5.30) | 20.20(5.09) | 20.71(5.11) ^b^ | 21.64(5.76) ^b^ | | <0.001 | |
| Missing | 2072(19.4) |  |  |  |  | |  | |
| Diabetes |  |  |  |  |  | |  | |
| Yes | 1638(15.4) | 1420(15.6) | 113(17.9) | 73(12.6) | 32(15.2) | | 0.091 | |
| No | 8896(83.5) | 7694(84.4) | 518(82.1) | 505(87.4) | 179(84.8) | |  | |
| Missing | 126(1.2) |  |  |  |  | |  | |
| Heart disease |  |  |  |  |  |  | |  |
| Yes | 2246(21.1) | 1913(21.0) | 156(24.6) | 119(20.5) | 58(27.0) | 0.027 | |  |
| No | 8308(77.9) | 7213(79.0) | 477(75.4) | 461(79.5) | 157(73.0) |  | |  |
| Missing | 106(1.0) |  |  |  |  |  | |  |
| Cerebrovascular disease | |  |  |  |  |  | |  |
| Yes | 1787(16.8) | 1523(16.7) | 126(19.9) | 92(15.8) | 46(21.7) | 0.040 | |  |
| No | 8779(82.4) | 7614(83.3) | 508(80.1) | 491(84.2) | 166(78.3) |  | |  |
| Missing | 94(0.9) |  |  |  |  |  | |  |
| Respiratory disease |  |  |  |  |  |  | |  |
| Yes | 2060(19.3) | 1798(19.7) | 133(21.1) | 82(14.1) ^a^ | 47(22.0) | 0.005 | |  |
| No | 8484(79.6) | 7320(80.3) | 498(78.9) | 499(85.9) | 167(78.0) |  | |  |
| Missing | 116(1.1) |  |  |  |  |  | |  |
| Cancer |  |  |  |  |  |  | |  |
| Yes | 882(8.3) | 768(8.5) | 64(10.2) | 34(5.9) | 16(7.6) | 0.053 | |  |
| No | 9573(89.8) | 8274(91.5) | 561(89.8) | 543(94.1) | 195(92.4) |  | |  |
| Missing | 205(1.9) |  |  |  |  |  | |  |

SBP, systolic blood pressure. Data are obtained at the third visit unless noted and expressed as numbers (percentages) or median (interquartile range). Class 1: normal SBP; class 2: stabilized SBP; class 3: elevated SBP; and class 4: persistently high SBP. ^a^ There are statistically significant differences in the pairwise comparison between this group and any other groups.
