# Supplementary material for: Trajectory patterns of blood pressure change up to six years and the risk of dementia: a nationwide cohort study
Source: Aging (Albany NY). 2021 Jul 1;13(13):17380–406. doi: 10.18632/aging.203228 (PMC8312414; doi:10.18632/aging.203228)
Supplement: Supplementary Tables 5 and 6 [file aging-13-203228-s004.pdf]

Supplementary Table 5. Demographic and clinical characteristics of CLHLS participants by DBP trajectory classes.

Characteristics	Overall (*n*=10660)	Class 1 (*n*=10324)	Class 2 (*n*=203)	Class 3 (*n*=133)	*P* value
80-115 years old [a]	6881(64.5)	6634(64.3)	143(70.4)	104(78.2) [b]	0.001
Female	5755(54.0)	5567(53.9)	116(57.1)	72(54.1)	0.660
Han nationality	9916(93.0)	9600(93.0)	190(93.6)	126(94.7)	0.696
Education					
No schooling	6008(56.4)	5816(56.3)	114(56.2)	78(58.6)	0.575
Primary school	3439(32.3)	3328(32.2)	65(32.0)	46(34.6)	
White-collar	964(9.0)	937(9.1)	21(10.3)	6(4.5)	0.153
Average household income (yuan)					
< 5000	5020(47.1)	4837(46.9)	109(53.7)	74(55.6)	0.002
5000-19999	3955(37.1)	3836(37.2)	66(32.5)	53(39.8)	
Place of residence					
City	2294(21.5)	2224(21.5)	50(24.6)	20(15.0)	0.173
Town	3353(31.5)	3257(31.5)	54(26.6)	42(31.6)	
Smoking status					
Current	1695(15.9)	1653(16.0)	21(10.3)	21(15.8)	0.165
Past	2076(19.5)	2009(19.5)	46(22.7)	21(15.8)	
Alcohol use					
Current	1715(16.1)	1669(16.2)	20(9.9)	26(19.5)	0.043
Past	1762(16.5)	1694(16.4)	43(21.2)	25(18.8)	
Regular exercise					
Current	3068(28.8)	2973(28.8)	57(28.1)	38(28.6)	0.263
Past	1811(17.0)	1739(16.8)	45(22.2)	27(20.3)	
Sleep quality					
Very good or good	6240(58.5)	6041(58.5)	117(57.6)	82(61.7)	0.679
Fair	2987(28.0)	2898(28.1)	59(29.1)	30(22.6)	
Sleep duration (hours)	8.00(4.00)	8.00(4.00)	8.00(4.00)	8.00(4.00)	0.305
Living alone	1621(15.2)	1574(15.2)	30(14.8)	17(12.8)	0.723
Heart rate (beat/ minute)	73(12)	73(12)	72(12)	75(15)	0.189
Body mass index (kg/m^2)	20.05(5.31)	20.05(5.31)	20.00(4.94)	20.00(4.98)	0.877
Diabetes	1659(15.6)	1620(15.7)	21(10.3)	18(13.5)	0.093
Heart disease	2272(21.3)	2184(21.2)	57(28.1)	31(23.3)	0.050
Cerebrovascular disease	1800(16.9)	1736(16.8)	38(18.7)	26(19.5)	0.550
Respiratory disease	2076(19.5)	2021(19.6)	28(13.8)	27(20.3)	0.116
Cancer	900(8.4)	880(8.5)	8(3.9)	12(9.0)	0.065
Dementia	1049(9.8)	1021(9.9)	14(6.9)	14(10.5)	0.353

DBP, diastolic blood pressure. Data are obtained at the third visit unless noted and expressed as numbers (percentages) or median (interquartile range). Class 1, normal DBP; class 2, stabilized DBP; class 3, elevated DBP.

[a]Obtained at the first visit.

[b]There are statistically significant differences in the pairwise comparison between this class and class 1 trajectory.

Supplementary Table 6. Demographic and clinical characteristics of CLHLS participants by PP trajectory classes.

Characteristics	Class 1 (*n*=8889)	Class 2 (*n*=904)	Class 3 (*n*=683)	Class 4 (*n*=184)	*P* value
80-115 years old [a]	5641(63.5)	708(78.3) [b]	410(60.0)	122(66.3)	<0.001
Female	4714(53.0)	527(58.3) [b]	395(57.8)	119(64.7) [b]	<0.001
Han nationality	8260(92.9)	848(93.8)	638(93.4)	170(92.4)	0.739
Education					
No schooling	4940(55.6)	555(61.4) [b]	407(59.6)	106(57.6)	0.020
Primary school	2916(32.8)	262(29.0)	202(29.6)	59(32.1)	
White-collar	811(9.1)	83(9.2)	49(7.2)	21(11.4)	0.236
Average household income (yuan)					
< 5000	4207(47.3)	457(50.6)	285(41.7) [b]	71(38.6)	0.003
5000-19999	3283(36.9)	323(35.7)	274(40.1)	75(40.8)	
Place of residence					
City	1940(21.8)	208(23.0)	112(16.4) [b]	34(18.5)	<0.001
Town	2819(31.7)	275(30.4)	223(32.7)	36(19.6) [b]	
Smoking status					
Current	1442(16.2)	121(13.4)	109(16.0)	23(12.5)	0.064
Past	1748(19.7)	160(17.7)	137(20.1)	31(16.8)	
Alcohol use					
Current	1447(16.3)	132(14.6)	111(16.3)	25(13.6)	0.002
Past	1511(17.0)	138(15.3)	99(14.5)	14(7.6)	
Regular exercise					
Current	2605(29.3)	227(25.1) [b]	188(27.5)	48(26.1)	0.004
Past	1526(17.2)	168(18.6)	92(13.5)	25(13.6)	
Sleep quality					
Very good or good	5187(58.4)	528(58.4)	422(61.8)	103(56.0)	0.173
Fair	2515(28.3)	259(28.7)	159(23.3)	54(29.3)	
Sleep duration (hours)	8.00(4.00)	8.00(4.00)	8.00(4.00)	8.00(4.00)	0.772
Living alone	1330(15.0)	156(17.3)	105(15.4)	30(16.3)	0.315
Heart rate (beat/ minute)	73(12)	72(12)	73(14)	74(13)	0.057
Body mass index (kg/m^2)	20.00(5.26)	20.20(5.19)	20.41(5.64) [b]	21.26(6.34) [b]	<0.001
Diabetes	1381(15.5)	162(17.9)	83(12.2)	33(17.9)	0.014
Heart disease	1864(21.0)	225(24.9) [b]	138(20.2)	45(24.5)	0.028
Cerebrovascular disease	1496(16.8)	174(19.2)	99(14.5)	31(16.8)	0.094
Respiratory disease	1765(19.9)	188(20.8)	86(12.6) [b]	37(20.1)	<0.001
Cancer	752(8.5)	95(10.5)	39(5.7)	14(7.6)	0.008
Dementia	887(10.0)	106(11.7)	43(6.3) [b]	13(7.1)	0.002

PP, pulse pressure. Data are obtained at the third visit unless noted and expressed as numbers (percentages) or median (interquartile range). Class 1, normal PP; class 2, stabilized PP; class 3, elevated PP; and class 4, persistently high PP.

[a]Obtained at the first visit.

[b]There are statistically significant differences in the pairwise comparison between this class and class 1 trajectory.
