# Supplementary material for: Trajectory patterns of blood pressure change up to six years and the risk of dementia: a nationwide cohort study
Source: Aging (Albany NY). 2021 Jul 1;13(13):17380–406. doi: 10.18632/aging.203228 (PMC8312414; doi:10.18632/aging.203228)
Supplement: Supplementary Table 7 [file aging-13-203228-s005.docx]

**Supplementary Table 7. Effects of PP trajectory on the risk of dementia in different subgroups.**

| **Variables** | **Model 1** | **Model 2** | **Model 3** | **Model 4** |
| --- | --- | --- | --- | --- |
| 60-79 years old at the first visit | |  |  |  |
| **Normal PP as reference** | |  |  |  |
| Stabilized PP | 0.84(0.48, 1.46) | 0.81(0.46, 1.41) | 0.83(0.48, 1.45) | 0.92(0.51, 1.63) |
| Elevated PP | 0.57(0.33, 0.99) * | 0.58(0.33, 1.00) | 0.59(0.34, 1.03) | 1.04(0.58, 1.86) |
| Persistently high PP | 0.59(0.22, 1.58) | 0.65(0.24, 1.75) | 0.69(0.26, 1.86) | 1.03(0.37, 2.86) |
| **Persistently high PP as reference** | |  |  |  |
| Stabilized PP | 1.42(0.46, 4.36) | 1.24(0.40, 3.81) | 1.21(0.39, 3.73) | 0.89(0.28, 2.81) |
| Elevated PP | 0.96(0.31, 2.95) | 0.88(0.29, 2.72) | 0.85(0.28, 2.63) | 1.00(0.32, 3.14) |
| **Elevated PP as reference** | |  |  |  |
| Normal PP | 1.76(1.01, 3.07) * | 1.74(1.00, 3.04) | 1.70(0.98, 2.97) | 0.97(0.54, 1.73) |
| Stabilized PP | 1.48(0.68, 3.19) | 1.40(0.65, 3.04) | 1.42(0.65, 3.07) | 0.88(0.40, 1.97) |
| 80-115 years old at the first visit | |  |  |  |
| **Normal PP as reference** | |  |  |  |
| Stabilized PP | 1.72(1.38, 2.15) *** | 1.83(1.47, 2.28) *** | 1.87(1.50, 2.33) *** | 1.59(1.27, 2.00) *** |
| Elevated PP | 0.56(0.39, 0.81) ** | 0.59(0.41, 0.84) ** | 0.59(0.41, 0.86) * | 0.68(0.47, 0.99) * |
| Persistently high PP | 0.68(0.35, 1.31) | 0.68(0.35, 1.31) | 0.70(0.36, 1.36) | 0.84(0.43, 1.62) |
| **Persistently high PP as reference** | |  |  |  |
| Stabilized PP | 2.55(1.28, 5.05) ** | 2.69(1.36, 5.35) ** | 2.66(1.34, 5.28) ** | 1.91(0.95, 3.82) |
| Elevated PP | 0.83(0.39, 1.74) | 0.86(0.41, 1.81) | 0.85(0.40, 1.78) | 0.82(0.39, 1.74) |
| **Elevated PP as reference** | |  |  |  |
| Normal PP | 1.79(1.24, 2.59) ** | 1.71(1.18, 2.47) ** | 1.69(1.17, 2.44) ** | 1.46(1.01, 2.13) * |
| Stabilized PP | 3.09(2.04, 4.66) *** | 3.13(2.07, 4.73) *** | 3.14(2.08, 4.76) *** | 2.33(1.53, 3.55) *** |
| Male |  |  |  |  |
| **Normal PP as reference** | |  |  |  |
| Stabilized PP | 1.47(1.07, 2.01) * | 1.43(1.04, 1.95) * | 1.44(1.05, 1.98) * | 1.45(1.04, 2.02) * |
| Elevated PP | 0.57(0.35, 0.91) * | 0.56(0.35, 0.89) * | 0.56(0.35, 0.89) * | 0.98(0.60, 1.60) |
| Persistently high PP | 0.70(0.29, 1.68) | 0.58(0.24, 1.41) | 0.59(0.24, 1.43) | 0.90(0.37, 2.20) |
| **Persistently high PP as reference** | |  |  |  |
| Stabilized PP | 2.11(0.84, 5.33) | 2.45(0.97, 6.20) | 2.44(0.96, 6.19) | 1.61(0.63, 4.12) |
| Elevated PP | 0.81(0.30, 2.19) | 0.96(0.35, 2.58) | 0.94(0.35, 2.54) | 1.09(0.40, 2.98) |
| **Elevated** **PP as reference** | |  |  |  |
| Normal PP | 1.77(1.10, 2.83) * | 1.80(1.12, 2.88) * | 1.80(1.12, 2.89) * | 1.02(0.62, 1.66) |
| Stabilized PP | 2.59(1.50, 4.50) ** | 2.56(1.48, 4.45) ** | 2.60(1.50, 4.51) ** | 1.48(0.83, 2.62) |
| Female |  |  |  |  |
| **Normal PP as reference** | |  |  |  |
| Stabilized PP | 1.86(1.43, 2.42) *** | 1.79(1.37, 2.34) *** | 1.84(1.41, 2.41) *** | 1.66(1.26, 2.19) *** |
| Elevated PP | 0.57(0.38, 0.85) ** | 0.58(0.39, 0.87) ** | 0.59(0.39, 0.88) * | 0.56(0.37, 0.85) ** |
| Persistently high PP | 0.67(0.34, 1.36) | 0.78(0.39, 1.57) | 0.80(0.40, 1.61) | 0.87(0.43, 1.78) |
| **Persistently high PP as reference** | |  |  |  |
| Stabilized PP | 2.76(1.32, 5.75) ** | 2.31(1.10, 4.82) * | 2.31(1.10, 4.84) * | 1.91(0.90, 4.04) |
| Elevated PP | 0.84(0.38, 1.86) | 0.75(0.34, 1.66) | 0.74(0.33, 1.64) | 0.65(0.29, 1.46) |
| **Elevated PP as reference** | |  |  |  |
| Normal PP | 1.77(1.18, 2.64) ** | 1.72(1.15, 2.58) ** | 1.70(1.14, 2.55) * | 1.78(1.17, 2.70) ** |
| Stabilized PP | 3.28(2.06, 5.22) *** | 3.08(1.94, 4.91) *** | 3.14(1.97, 5.00) *** | 2.96(1.83, 4.79) *** |
| Hypertension at the first visit | |  |  |  |
| **Normal PP as reference** | |  |  |  |
| Stabilized PP | 2.00(1.46, 2.74) *** | 2.15(1.57, 2.96) *** | 2.20(1.60, 3.03) *** | 3.00(2.12, 4.24) *** |
| Elevated PP | 0.69(0.41, 1.17) | 0.72(0.43, 1.23) | 0.76(0.45, 1.29) | 0.96(0.55, 1.68) |
| Persistently high PP | 0.84(0.41, 1.70) | 0.91(0.45, 1.84) | 0.94(0.46, 1.92) | 0.98(0.47, 2.04) |
| **Persistently high PP as reference** | |  |  |  |
| Stabilized PP | 2.39(1.13, 5.04) * | 2.38(1.12, 5.03) * | 2.33(1.10, 4.95) * | 3.06(1.41, 6.66) ** |
| Elevated PP | 0.82(0.35, 1.94) | 0.80(0.34, 1.89) | 0.80(0.34, 1.90) | 0.98(0.41, 2.38) |
| **Elevated PP as reference** | |  |  |  |
| Normal PP | 1.45(0.86, 2.46) | 1.38(0.82, 2.35) | 1.32(0.78, 2.24) | 1.04(0.60, 1.81) |
| Stabilized PP | 2.90(1.63, 5.17) *** | 2.98(1.67, 5.32) *** | 2.90(1.62, 5.19) | 3.11(1.69, 5.72) *** |
| Non-hypertension at the first visit | |  |  |  |
| **Normal PP as reference** | |  |  |  |
| Stabilized PP | 1.62(1.23, 2.13) ** | 1.49(1.14, 1.96) ** | 1.53(1.16, 2.01) ** | 1.32(1.00, 1.75) |
| Elevated PP | 0.53(0.36, 0.77) ** | 0.53(0.36, 0.77) ** | 0.53(0.36, 0.77) ** | 0.62(0.42, 0.91) * |
| Persistently high PP | 0.60(0.25, 1.46) | 0.59(0.25, 1.43) | 0.62(0.26, 1.50) | 1.10(0.46, 2.68) |
| **Persistently high PP as reference** | |  |  |  |
| Stabilized PP | 2.68(1.07, 6.70) * | 2.53(1.01, 6.32) * | 2.46(0.98, 6.16) | 1.20(0.47, 3.01) |
| Elevated PP | 0.87(0.34, 2.26) | 0.89(0.34, 2.31) | 0.85(0.33, 2.20) | 0.56(0.22, 1.47) |
| **Elevated PP as reference** | |  |  |  |
| Normal PP | 1.90(1.30, 2.78) ** | 1.90(1.30, 2.77) ** | 1.90(1.30, 2.77) ** | 1.61(1.10, 2.37) * |
| Stabilized PP | 3.08(1.96, 4.85) *** | 2.84(1.80, 4.47) *** | 2.91(1.84, 4.57) *** | 2.13(1.34, 3.38) ** |

PP, pulse pressure. Hazard ratios (95% confidence intervals) are presented. Model 1 was adjusted for no covariates. Model 2 was adjusted for age, gender, ethnic group, education, primary occupation before retirement, average household income, and place of residence. Model 3 was adjusted for model 2 plus smoking, alcohol use, regular exercise, sleep quality, sleep duration, and living alone. Model 4 was adjusted for model 3 plus heart rate, body mass index, hypertension, diabetes, heart disease, cerebrovascular disease, respiratory disease, and cancer. * *P* <0.05, ** *P* < 0.01, *** *P* < 0.001.
