# Supplementary material for: Trajectory patterns of blood pressure change up to six years and the risk of dementia: a nationwide cohort study
Source: Aging (Albany NY). 2021 Jul 1;13(13):17380–406. doi: 10.18632/aging.203228 (PMC8312414; doi:10.18632/aging.203228)
Supplement: Supplementary Tables 8 to 12 [file aging-13-203228-s006.pdf]

Supplementary Table 8. Sensitivity analyses on the effects of SBP trajectory on the risk of dementia.

Variables	Model 1	Model 2	Model 3	Model 4
Excluding 1141 participants with a history of heart disease at the first visit				
**Normal SBP as reference**				
Stabilized SBP	1.66(1.28, 2.15) ***	1.65(1.28, 2.13) ***	1.70(1.32, 2.20) ***	1.44(1.10, 1.88) **
Elevated SBP	0.60(0.42, 0.84) **	0.65(0.46, 0.92) *	0.65(0.46, 0.92) *	0.69(0.48, 0.98) *
Persistently high SBP	0.94(0.56, 1.56)	0.96(0.58, 1.61)	0.97(0.58, 1.61)	1.09(0.64, 1.84)
**Persistently high SBP as reference**				
Stabilized SBP	1.78(1.01, 3.12) *	1.71(0.97, 3.01)	1.76(1.00, 3.10) *	1.32(0.74, 2.36)
Elevated SBP	0.64(0.35, 1.17)	0.68(0.37, 1.25)	0.67(0.36, 1.24)	0.63(0.34, 1.17)
**Elevated SBP as reference**				
Normal SBP	1.68(1.19, 2.38) **	1.54(1.08, 2.18) *	1.54(1.09, 2.19) *	1.46(1.02, 2.08) *
Stabilized SBP	2.79(1.83, 4.25) ***	2.54(1.66, 3.87) ***	2.63(1.72, 4.01) ***	2.10(1.36, 3.22) **
Excluding 523 participants with a history of diabetes at the first visit				
**Normal SBP as reference**				
Stabilized SBP	1.85(1.45, 2.34) ***	1.85(1.46, 2.35) ***	1.90(1.50, 2.42) ***	1.59(1.24, 2.04) ***
Elevated SBP	0.66(0.48, 0.91) *	0.73(0.53, 1.00)	0.73(0.53, 1.00)	0.75(0.54, 1.03)
Persistently high SBP	1.05(0.66, 1.65)	1.11(0.70, 1.75)	1.12(0.71, 1.76)	1.07(0.67, 1.72)
**Persistently high SBP as reference**				
Stabilized SBP	1.77(1.07, 2.92) *	1.67(1.01, 2.77) *	1.70(1.03, 2.82) *	1.48(0.88, 2.49)
Elevated SBP	0.63(0.37, 1.10)	0.65(0.38, 1.13)	0.65(0.37, 1.12)	0.69(0.40, 1.21)
**Elevated SBP as reference**				
Normal SBP	1.51(1.10, 2.08) *	1.38(1.00, 1.90)	1.38(1.00, 1.90)	1.34(0.97, 1.86)
Stabilized SBP	2.79(1.89, 4.11) ***	2.56(1.73, 3.77) ***	2.63(1.78, 3.88) ***	2.14(1.44, 3.17) ***
Excluding 619 participants at the diagnosis of cerebrovascular disease at the first visit				
**Normal SBP as reference**				
Stabilized SBP	1.81(1.42, 2.30) ***	1.80(1.42, 2.30) ***	1.84(1.45, 2.34) ***	1.57 (1.22, 2.01) **
Elevated SBP	0.63(0.46, 0.88) **	0.69(0.50, 0.96) *	0.69(0.49, 0.96) *	0.73(0.52, 1.02)
Persistently high SBP	1.07(0.67, 1.70)	1.10(0.69, 1.76)	1.10(0.69, 1.76)	1.10(0.68, 1.78)
**Persistently high SBP as reference**				
Stabilized SBP	1.70(1.02, 2.85)	1.64(0.98, 2.75)	1.67(1.00, 2.80)	1.43(0.84, 2.44)
Elevated SBP	0.60(0.34, 1.05)	0.63(0.36, 1.10)	0.62(0.35, 1.10)	0.66(0.37, 1.18)
**Elevated SBP as reference**				
Normal SBP	1.58(1.14, 2.19) **	1.45(1.04, 2.02) *	1.46(1.05, 2.03) *	1.38(0.98, 1.93)
Stabilized SBP	2.86(1.92, 4.25) ***	2.61(1.76, 3.89) ***	2.68(1.80, 3.99) ***	2.16(1.44, 3.22) ***
Excluding 192 participants screened as moderate or severe cognitive impairment at the first visit				
**Normal SBP as reference**				
Stabilized SBP	1.75(1.38, 2.21) ***	1.76(1.39, 2.23) ***	1.80(1.41, 2.28) ***	1.61(1.26, 2.06) ***
Elevated SBP	0.63(0.46, 0.87) **	0.69(0.50, 0.95) *	0.69(0.50, 0.95) *	0.72(0.52, 0.99) *
Persistently high SBP	0.94(0.59, 1.51)	1.02(0.64, 1.63)	1.02(0.64, 1.63)	1.20(0.74, 1.93)
**Persistently high SBP as reference**				
Stabilized SBP	1.85(1.10, 3.09)	1.73(1.03, 2.89)	1.75(1.05, 2.94) *	1.35(0.80, 2.27)
Elevated SBP	0.67(0.38, 1.16)	0.68(0.39, 1.18)	0.67(0.38, 1.17)	0.60(0.34, 1.06)
**Elevated SBP as reference**				
Normal SBP	1.59(1.15, 2.19) *	1.45(1.06, 2.01) *	1.46(1.06, 2.01) *	1.40(1.01, 1.93) *
Stabilized SBP	2.77(1.88, 4.09) ***	2.56(1.73, 3.77) ***	2.62(1.77, 3.86) ***	2.25(1.51, 3.33) ***

SBP, systolic blood pressure. Hazard ratios (95% confidence intervals) are presented. Model 1 was adjusted for no covariates. Model 2 was adjusted for age, gender, ethnic group, education, primary occupation before retirement, average household income, and place of residence. Model 3 was adjusted for model 2 plus smoking, alcohol use, regular exercise, sleep quality,

sleep duration, and living alone. Model 4 was adjusted for model 3 plus heart rate, body mass index, hypertension, diabetes, heart disease, cerebrovascular disease, respiratory disease, and cancer. *P <0.05, ** P < 0.01, *** P < 0.001.

Supplementary Table 9. Effects of DBP trajectory on the risk of dementia.

Variables	Model 1	Model 2	Model 3	Model 4
**Normal DBP as reference**				
Stabilized DBP	0.80(0.47, 1.35)	0.76(0.45, 1.29)	0.76(0.45, 1.29)	1.19(0.70, 2.03)
Elevated DBP	1.48(0.88, 2.52)	1.49(0.88, 2.53)	1.42(0.84, 2.42)	0.99(0.57, 1.71)
**Elevated DBP as reference**				
Normal DBP	0.67(0.40, 1.14)	0.67(0.40, 1.14)	0.70(0.41, 1.19)	1.01(0.59, 1.74)
Stabilized DBP	0.54(0.26, 1.12)	0.51(0.24, 1.07)	0.53(0.25, 1.12)	1.20(0.56, 2.57)

DBP, diastolic blood pressure. Hazard ratios (95% confidence intervals) are presented. Model 1 was adjusted for no covariates. Model 2 was adjusted for age, gender, ethnic group, education, primary occupation before retirement, average household income, and place of residence. Model 3 was adjusted for model 2 plus smoking, alcohol use, regular exercise, sleep quality, sleep duration, and living alone. Model 4 was adjusted for model 3 plus heart rate, body mass index, hypertension, diabetes, heart disease, cerebrovascular disease, respiratory disease, and cancer.

Supplementary Table 10. Sensitivity analyses on the effects of DBP trajectory on the risk of dementia.

Variables	Model 1	Model 2	Model 3	Model 4
Excluding 1141 participants with a history of heart disease at the first visit				
**Normal DBP as reference**				
Stabilized DBP	0.70(0.38, 1.31)	0.69(0.37, 1.28)	0.69(0.37, 1.29)	1.17(0.62, 2.21)
Elevated DBP	1.32(0.73, 2.40)	1.29(0.71, 2.34)	1.24(0.68, 2.24)	1.02(0.55, 1.89)
**Elevated DBP as reference**				
Normal DBP	0.76(0.42, 1.37)	0.78(0.42, 1.41)	0.81(0.45, 1.47)	0.98(0.53, 1.83)
Stabilized DBP	0.53(0.23, 1.25)	0.53(0.23, 1.26)	0.56(0.24, 1.31)	1.15(0.47, 2.80)
Excluding 523 participants with a history of diabetes at the first visit				
**Normal DBP as reference**				
Stabilized DBP	0.80(0.46, 1.39)	0.77(0.45, 1.34)	0.77(0.45, 1.34)	1.11(0.64, 1.93)
Elevated DBP	1.40(0.81, 2.43)	1.42(0.82, 2.46)	1.35(0.78, 2.34)	0.98(0.56, 1.73)
**Elevated DBP as reference**				
Normal DBP	0.71(0.41, 1.23)	0.70(0.41, 1.22)	0.74(0.43, 1.28)	1.02(0.58, 1.80)
Stabilized DBP	0.57(0.27, 1.23)	0.54(0.25, 1.17)	0.57(0.26, 1.23)	1.13(0.51, 2.51)
Excluding 619 participants at the diagnosis of cerebrovascular disease at the first visit				
**Normal DBP as reference**				
Stabilized DBP	0.71(0.39, 1.29)	0.68(0.37, 1.22)	0.68(0.37, 1.23)	1.10(0.60, 2.01)
Elevated DBP	1.59(0.94, 2.70)	1.60(0.94, 2.72)	1.51(0.89, 2.57)	0.99(0.57, 1.72)
**Elevated DBP as reference**				
Normal DBP	0.63(0.37, 1.06)	0.63(0.37, 1.06)	0.66(0.39, 1.13)	1.01(0.58, 1.74)
Stabilized DBP	0.45(0.20, 0.98) *	0.42(0.19, 0.93) *	0.45(0.20, 0.99) *	1.11(0.49, 2.51)
Excluding 192 participants screened as moderate or severe cognitive impairment at the first visit				
**Normal DBP as reference**				
Stabilized DBP	0.75(0.44, 1.30)	0.72(0.42, 1.25)	0.72(0.42, 1.25)	1.17(0.67, 2.04)
Elevated DBP	1.50(0.88, 2.53)	1.50(0.88, 2.54)	1.43(0.84, 2.43)	1.00(0.58, 1.73)
**Elevated DBP as reference**				
Normal DBP	0.67(0.40, 1.13)	0.67(0.39, 1.13)	0.70(0.41, 1.19)	1.00(0.58, 1.72)
Stabilized DBP	0.50(0.24, 1.07)	0.48(0.23, 1.03)	0.50(0.24, 1.07)	1.17(0.54, 2.54)

DBP, diastolic blood pressure. Hazard ratios (95% confidence intervals) are presented. Model 1 was adjusted for no covariates. Model 2 was adjusted for age, gender, ethnic group, education, primary occupation before retirement, average household income, and place of residence. Model 3 was adjusted for model 2 plus smoking, alcohol use, regular exercise, sleep quality, sleep duration, and living alone. Model 4 was adjusted for model 3 plus heart rate, body mass index, hypertension, diabetes, heart disease, cerebrovascular disease, respiratory disease, and cancer. **P* <0.05.

Supplementary Table 11. Effects of DBP trajectory on the risk of dementia in different subgroups.

Variables	Model 1	Model 2	Model 3	Model 4
60-79 years old at the first visit				
**Normal DBP as reference**				
Stabilized DBP	1.25(0.56, 2.81)	1.34(0.60, 3.02)	1.37(0.61, 3.08)	1.70(0.73, 3.96)
Elevated SBP	0.45(0.06, 3.22)	0.44(0.06, 3.16)	0.42(0.06, 2.99)	0.32(0.04, 2.39)
**Elevated DBP as reference**				
Normal DBP	2.26(0.32, 16.10)	2.26(0.32, 16.08)	2.40(0.34, 17.16)	3.09(0.42, 22.80)
Stabilized DBP	2.37(0.28, 20.32)	3.03(0.36, 25.21)	3.27(0.39, 27.38)	5.24(0.61, 44.94)
80-115 years old at the first visit				
**Normal DBP as reference**				
Stabilized DBP	0.60(0.30, 1.20)	0.59(0.29, 1.18)	0.59(0.29, 1.18)	1.01(0.50, 2.06)
Elevated DBP	1.58(0.91, 2.73)	1.68(0.97, 2.92)	1.63(0.94, 2.82)	1.27(0.72, 2.24)
**Elevated DBP as reference**				
Normal DBP	0.63(0.37, 1.10)	0.59(0.34, 1.03)	0.62(0.35, 1.07)	0.79(0.47, 1.40)
Stabilized DBP	0.38(0.16, 0.92) *	0.35(0.15, 0.85) *	0.36(0.15, 0.88) *	0.80(0.32, 1.98)
Male				
**Normal DBP as reference**				
Stabilized DBP	0.75(0.34, 1.69)	0.67(0.30, 1.50)	0.66(0.29, 1.48)	0.97(0.43, 2.22)
Elevated DBP	1.31(0.59, 2.93)	1.43(0.64, 3.20)	1.35(0.60, 3.02)	1.19(0.51, 2.76)
**Elevated DBP as reference**				
Normal DBP	0.76(0.34, 1.71)	0.70(0.31, 1.57)	0.74(0.33, 1.67)	0.84(0.36, 1.95)
Stabilized DBP	0.58(0.19, 1.78)	0.47(0.15, 1.45)	0.49(0.16, 1.52)	0.82(0.26, 2.59)
Female				
**Normal DBP as reference**				
Stabilized DBP	0.84(0.42, 1.68)	0.85(0.42, 1.70)	0.87(0.43, 1.75)	1.46(0.71, 3.02)
Elevated DBP	1.66(0.83, 3.34)	1.59(0.79, 3.21)	1.55(0.77, 3.13)	0.83(0.39, 1.75)
**Elevated DBP as reference**				
Normal DBP	0.60(0.30, 1.21)	0.63(0.31, 1.26)	0.65(0.32, 1.31)	1.21(0.57, 2.57)
Stabilized DBP	0.50(0.19, 1.34)	0.53(0.20, 1.42)	0.56(0.21, 1.50)	1.77(0.63, 5.01)
Hypertension at the first visit				
**Normal DBP as reference**				
Stabilized DBP	0.98(0.50, 1.91)	0.95(0.49, 1.85)	0.93(0.47, 1.81)	1.91(0.95, 3.86)
Elevated DBP	2.87(1.35, 6.09) **	3.06(1.43, 6.54) **	3.07(1.43, 6.59) **	4.37(1.96, 9.73) ***
**Elevated DBP as reference**				
Normal DBP	0.35(0.16, 0.74) **	0.33(0.15, 0.70) **	0.33(0.15, 0.70) **	0.23(0.10, 0.51) ***
Stabilized DBP	0.34(0.13, 0.92) *	0.31(0.12, 0.84) *	0.30(0.11, 0.82) *	0.44(0.16, 1.23)
Non-hypertension at the first visit				
**Normal DBP as reference**				
Stabilized DBP	0.73(0.30, 1.76)	0.69(0.29, 1.66)	0.68(0.28, 1.65)	1.04(0.43, 2.56)
Elevated DBP	1.01(0.48, 2.13)	1.05(0.50, 2.21)	0.99(0.47, 2.09)	0.48(0.22, 1.04)
**Elevated DBP as reference**				
Normal DBP	0.99(0.47, 2.08)	0.95(0.45, 2.01)	1.01(0.48, 2.13)	2.08(0.96, 4.50)
Stabilized DBP	0.72(0.23, 2.26)	0.66(0.21, 2.07)	0.69(0.22, 2.17)	2.17(0.66, 7.10)

DBP, diastolic blood pressure. Hazard ratios (95% confidence intervals) are presented. Model 1 was adjusted for no covariates. Model 2 was adjusted for age, gender, ethnic group, education, primary occupation before retirement, average household income, and place of residence. Model 3 was adjusted for model 2 plus smoking, alcohol use, regular exercise, sleep quality, sleep duration, and living alone. Model 4 was adjusted for model 3 plus heart rate, body mass index, hypertension, diabetes, heart disease, cerebrovascular disease, respiratory disease, and cancer. *P <0.05, ** P < 0.01, *** P < 0.001.

Supplementary Table 12. Effects of PP trajectory on the risk of dementia.

Variables	Model 1	Model 2	Model 3	Model 4
**Normal PP as reference**				
Stabilized PP	1.67(1.37, 2.05) ***	1.63(1.33, 1.99) ***	1.65(1.35, 2.02) ***	1.52(1.24, 1.88) ***
Elevated PP	0.56(0.41, 0.76) ***	0.57(0.42, 0.77) ***	0.57(0.42, 0.78) ***	0.72(0.53, 0.98) *
Persistently high PP	0.67(0.39, 1.17)	0.70(0.40, 1.20)	0.71(0.41, 1.24)	0.87(0.50, 1.51)
**Persistently high PP as reference**				
Stabilized PP	2.48(1.40, 4.42) **	2.34(1.31, 4.16) **	2.31(1.30, 4.12) **	1.75(0.98, 3.14)
Elevated PP	0.83(0.45, 1.55)	0.82(0.44, 1.52)	0.80(0.43, 1.50)	0.83(0.44, 1.55)
**Elevated PP as reference**				
Normal PP	1.78(1.31, 2.42) ***	1.76(1.30, 2.39) ***	1.74(1.28, 2.37) ***	1.39(1.02, 1.90) *
Stabilized PP	2.98(2.09, 4.25) ***	2.86(2.00, 4.08) ***	2.88(2.02, 4.11) ***	2.12(1.48, 3.04) ***

PP, pulse pressure. Hazard ratios (95% confidence intervals) are presented. Model 1 was adjusted for no covariates. Model 2 was adjusted for age, gender, ethnic group, education, primary occupation before retirement, average household income, and place of residence. Model 3 was adjusted for model 2 plus smoking status, alcohol use, regular exercise, sleep quality, sleep duration, and living alone. Model 4 was adjusted for model 3 plus heart rate, body mass index, hypertension, diabetes, heart disease, cerebrovascular disease, respiratory disease, and cancer. *P <0.05, ** P < 0.01, *** P < 0.001.
