# Supplementary material for: Trajectory patterns of blood pressure change up to six years and the risk of dementia: a nationwide cohort study
Source: Aging (Albany NY). 2021 Jul 1;13(13):17380–406. doi: 10.18632/aging.203228 (PMC8312414; doi:10.18632/aging.203228)
Supplement: Supplementary Table 13 [file aging-13-203228-s007.docx]

**Supplementary Table 13. Effects of SBP trajectory on the risk of dementia in different subgroups.**

| **Variables** | **Model 1** | **Model 2** | **Model 3** | **Model 4** |
| --- | --- | --- | --- | --- |
| 60-79 years old at the first visit | |  |  |  |
| **Normal SBP as reference** | |  |  |  |
| Stabilized SBP | 0.46(0.21, 1.04) | 0.48(0.22, 1.09) | 0.50(0.22, 1.12) | 0.55(0.24, 1.29) |
| Elevated SBP | 0.68(0.40, 1.17) | 0.74(0.43, 1.27) | 0.74(0.43, 1.28) | 0.91(0.52, 1.58) |
| Persistently high SBP | 0.90(0.40, 2.01) | 0.95(0.42, 2.13) | 0.97(0.43, 2.18) | 1.35(0.57, 3.17) |
| **Persistently high SBP as reference** | |  |  |  |
| Stabilized SBP | 0.52(0.17, 1.60) | 0.51(0.16, 1.58) | 0.51(0.16, 1.60) | 0.41(0.13, 1.33) |
| Elevated SBP | 0.76(0.29, 1.98) | 0.78(0.30, 2.04) | 0.77(0.29, 2.02) | 0.67(0.25, 1.83) |
| **Elevated SBP as reference** | |  |  |  |
| Normal SBP | 1.47(0.86, 2.50) | 1.35(0.79, 2.31) | 1.34(0.78, 2.30) | 1.10(0.63, 1.93) |
| Stabilized SBP | 0.68(0.26, 1.77) | 0.65(0.25, 1.70) | 0.67(0.26, 1.74) | 0.61(0.23, 1.64) |
| 80-115 years old at the first visit | |  |  |  |
| **Normal SBP as reference** | |  |  |  |
| Stabilized SBP | 2.07(1.61, 2.66) *** | 2.15(1.67, 2.76) *** | 2.19 (1.70, 2.82) *** | 1.87(1.44, 2.43) *** |
| Elevated SBP | 0.64(0.43, 0.94) * | 0.69(0.46, 1.02) | 0.69(0.47, 1.02) | 0.70(0.47, 1.04) |
| Persistently high SBP | 1.03(0.60, 1.79) | 1.07(0.61, 1.85) | 1.10(0.63, 1.90) | 1.13(0.64, 1.99) |
| **Persistently high SBP as reference** | |  |  |  |
| Stabilized SBP | 2.01(1.11, 3.63) * | 2.01(1.11, 3.65) * | 2.00(1.10, 3.63) * | 1.66(0.90, 3.04) |
| Elevated SBP | 0.62(0.32, 1.20) | 0.64(0.33, 1.26) | 0.63(0.32, 1.23) | 0.62(0.31, 1.22) |
| **Elevated SBP as reference** | |  |  |  |
| Normal SBP | 1.58(1.06, 2.33) * | 1.46(0.98, 2.16) | 1.45(0.98, 2.15) | 1.43(0.96, 2.14) |
| Stabilized SBP | 3.26(2.07, 5.12) *** | 3.12(1.99, 4.91) *** | 3.17(2.02, 5.00) *** | 2.67(1.69, 4.26) *** |
| Male |  |  |  |  |
| **Normal SBP as reference** | |  |  |  |
| Stabilized SBP | 1.49(1.01, 2.19) | 1.44(0.98, 2.13) | 1.50(1.01, 2.21) * | 1.32(0.87, 2.00) |
| Elevated SBP | 0.63(0.39, 1.04) | 0.68(0.41, 1.11) | 0.67(0.41, 1.11) | 0.83(0.50, 1.39) |
| Persistently high SBP | 1.10(0.57, 2.12) | 1.18(0.61, 2.28) | 1.15(0.60, 2.24) | 1.22(0.60, 2.47) |
| **Persistently high SBP as reference** | |  |  |  |
| Stabilized SBP | 1.36(0.64, 2.89) | 1.23(0.58, 2.62) | 1.30(0.61, 2.77) | 1.08(0.49, 2.42) |
| Elevated SBP | 0.58(0.26, 1.31) | 0.57(0.25, 1.30) | 0.58(0.26, 1.33) | 0.69(0.29, 1.61) |
| **Elevated SBP as reference** | |  |  |  |
| Normal SBP | 1.58(0.96, 2.60) | 1.48(0.90, 2.44) | 1.49(0.90, 2.45) | 1.20(0.72, 2.00) |
| Stabilized SBP | 2.35(1.26, 4.36) ** | 2.14(1.15, 3.97) * | 2.22(1.19, 4.14) * | 1.58(0.83, 3.01) |
| Female |  |  |  |  |
| **Normal SBP as reference** | |  |  |  |
| Stabilized SBP | 1.95(1.45, 2.63) *** | 1.97(1.46, 2.66) *** | 2.03(1.51, 2.74) *** | 1.98(1.45, 2.70) *** |
| Elevated SBP | 0.65(0.43, 0.98) * | 0.71(0.47, 1.08) | 0.71(0.47, 1.07) | 0.62(0.40, 0.95) * |
| Persistently high SBP | 0.88(0.47, 1.65) | 0.92(0.49, 1.73) | 0.92(0.49, 1.74) | 0.99(0.52, 1.89) |
| **Persistently high SBP as reference** | |  |  |  |
| Stabilized SBP | 2.21(1.12, 4.36) * | 2.14(1.08, 4.23) * | 2.20(1.11, 4.36) * | 2.00(0.99, 4.03) |
| Elevated SBP | 0.74(0.35, 1.54) | 0.78(0.37, 1.62) | 0.77(0.37, 1.61) | 0.62(0.29, 1.33) |
| **Elevated SBP as reference** | |  |  |  |
| Normal SBP | 1.54(1.02, 2.32) * | 1.40(0.93, 2.11) | 1.41(0.94, 2.14) | 1.62(1.06, 2.49) * |
| Stabilized SBP | 3.00(1.84, 4.90) *** | 2.76(1.69, 4.51) *** | 2.87(1.75, 4.70) *** | 3.21(1.93, 5.34) *** |
| Hypertension at the first visit | |  |  |  |
| **Normal SBP as reference** | |  |  |  |
| Stabilized SBP | 2.09(1.51, 2.90) *** | 2.31(1.66, 3.22) *** | 2.35(1.68, 3.28) *** | 2.94(2.03, 4.27) *** |
| Elevated SBP | 0.77(0.43, 1.37) | 0.90(0.50, 1.61) | 0.96(0.53, 1.73) | 1.79(0.96, 3.34) |
| Persistently high SBP | 1.12(0.61, 2.06) | 1.28(0.69, 2.37) | 1.36(0.73, 2.52) | 1.84(0.96, 3.51) |
| **Persistently high SBP as reference** | |  |  |  |
| Stabilized SBP | 1.87(0.97, 3.61) | 1.80(0.93, 3.50) | 1.73(0.89, 3.37) | 1.60(0.80, 3.21) |
| Elevated SBP | 0.68(0.30, 1.55) | 0.70(0.31, 1.59) | 0.71(0.31, 1.61) | 0.97(0.41, 2.29) |
| **Elevated SBP as reference** | |  |  |  |
| Normal SBP | 1.31(0.73, 2.34) | 1.12(0.62, 2.01) | 1.04(0.58, 1.88) | 0.56(0.30, 1.05) |
| Stabilized SBP | 2.73(1.44, 5.16) ** | 2.58(1.36, 4.88) ** | 2.45(1.29, 4.66) * | 1.65(0.83, 3.25) |
| Non-hypertension at the first visit | |  |  |  |
| **Normal SBP as reference** | |  |  |  |
| Stabilized SBP | 1.73(1.20, 2.49) ** | 1.61(1.12, 2.32) * | 1.67(1.16, 2.41) ** | 1.90(1.30, 2.76) ** |
| Elevated SBP | 0.61(0.42, 0.88) ** | 0.66(0.45, 0.97) * | 0.65(0.45, 0.96) * | 0.53(0.36, 0.78) ** |
| Persistently high SBP | 1.00(0.50, 2.01) | 1.04(0.52, 2.10) | 1.06(0.53, 2.14) | 0.98(0.48, 2.03) |
| **Persistently high SBP as reference** | |  |  |  |
| Stabilized SBP | 1.73(0.79, 3.77) | 1.54(0.71, 3.37) | 1.57(0.72, 3.44) | 1.93(0.86, 4.32) |
| Elevated SBP | 0.61(0.28, 1.33) | 0.63(0.29, 1.39) | 0.62(0.28, 1.36) | 0.54(0.24, 1.20) |
| **Elevated SBP as reference** | |  |  |  |
| Normal SBP | 1.65(1.13, 2.41) ** | 1.51(1.04, 2.21) ** | 1.53(1.05, 2.23) * | 1.90(1.29, 2.80) * |
| Stabilized SBP | 2.85(1.70, 4.77) *** | 2.43(1.45, 4.08) *** | 2.55(1.52, 4.28) ** | 3.60(2.12, 6.12) *** |

SBP, systolic blood pressure. Hazard ratios (95% confidence intervals) are presented. Model 1 was adjusted for no covariates. Model 2 was adjusted for age at the first visit, gender, ethnic group, education, primary occupation before retirement, average household income, and place of residence. Model 3 was adjusted for model 2 plus smoking, alcohol use, regular exercise, sleep quality, sleep duration, and living alone. Model 4 was adjusted for model 3 plus heart rate, body mass index, hypertension, diabetes, heart disease, cerebrovascular disease, respiratory disease, and cancer. * *P* <0.05, ** *P* < 0.01, *** *P* < 0.001.
