# Supplementary material for: Trajectory patterns of blood pressure change up to six years and the risk of dementia: a nationwide cohort study
Source: Aging (Albany NY). 2021 Jul 1;13(13):17380–406. doi: 10.18632/aging.203228 (PMC8312414; doi:10.18632/aging.203228)
Supplement: Supplementary Table 14 [file aging-13-203228-s008.pdf]

Supplementary Table 14. Sensitivity analyses on the effects of PP trajectory on the risk of dementia.

Variables	Model 1	Model 2	Model 3	Model 4
Excluding 1141 participants with a history of heart disease at the first visit				
**Normal PP as reference**				
Stabilized PP	1.58(1.27, 1.97) ***	1.50(1.21, 1.88) ***	1.54(1.23, 1.92) ***	1.34(1.07, 1.69) *
Elevated PP	0.48(0.34, 0.68) ***	0.48(0.34, 0.68) ***	0.48(0.34, 0.69) ***	0.69(0.48, 0.99) *
Persistently high PP	0.62(0.33, 1.15)	0.61(0.33, 1.14)	0.63(0.34, 1.17)	1.00(0.53, 1.88)
**Persistently high PP as reference**				
Stabilized PP	2.57(1.34, 4.95) **	2.46(1.28, 4.74) **	2.45(1.27, 4.72) **	1.34(0.69, 2.61)
Elevated PP	0.78(0.38, 1.59)	0.78(0.38, 1.59)	0.77(0.38, 1.56)	0.69(0.34, 1.41)
**Elevated PP as reference**				
Normal PP	2.09(1.47, 2.98) ***	2.09(1.47, 2.98) ***	2.08(1.46, 2.97) ***	1.45(1.01, 2.08) *
Stabilized PP	3.30(2.20, 4.95) ***	3.14(2.10, 4.72) ***	3.20(2.13, 4.80) ***	1.95(1.29, 2.94) **
Excluding 523 participants with a history of diabetes at the first visit				
**Normal PP as reference**				
Stabilized PP	1.73(1.41, 2.12) ***	1.69(1.37, 2.07) ***	1.71(1.39, 2.10) ***	1.52(1.23, 1.87) ***
Elevated PP	0.51(0.36, 0.71) ***	0.51(0.37, 0.72) ***	0.52(0.37, 0.72) ***	0.75(0.53, 1.05)
Persistently high PP	0.74(0.43, 1.29)	0.74(0.43, 1.28)	0.77(0.44, 1.33)	0.95(0.54, 1.65)
**Persistently high PP as reference**				
Stabilized PP	2.33(1.31, 4.14) **	2.28(1.28, 4.06) **	2.24(1.26, 3.99) **	1.60(0.89, 2.88)
Elevated PP	0.68(0.36, 1.29)	0.69(0.37, 1.31)	0.68(0.36, 1.28)	0.79(0.42, 1.49)
**Elevated PP as reference**				
Normal PP	1.97(1.41, 2.75) ***	1.95(1.40, 2.72) ***	1.93(1.38, 2.70) ***	1.34(0.96, 1.89)
Stabilized PP	3.41(2.33, 4.98) ***	3.29(2.25, 4.82) ***	3.31(2.26, 4.84) ***	2.04(1.38, 3.00) ***
Excluding 619 participants at the diagnosis of cerebrovascular disease at the first visit				
**Normal PP as reference**				
Stabilized PP	1.66(1.35, 2.05) ***	1.62(1.31, 2.00) ***	1.65(1.33, 2.03) ***	1.54(1.24, 1.91) ***
Elevated PP	0.54(0.39, 0.75) ***	0.55(0.40, 0.76) ***	0.55(0.40, 0.76) ***	0.73(0.53, 1.01)
Persistently high PP	0.76(0.44, 1.32)	0.76(0.44, 1.31)	0.78(0.45, 1.35)	0.97(0.56, 1.69)
**Persistently high** PP **as reference**				
Stabilized PP	2.18(1.22, 3.89) **	2.15(1.20, 3.84) *	2.12(1.19, 3.79) *	1.59(0.88, 2.86)
Elevated PP	0.71(0.38, 1.33)	0.73(0.39, 1.36)	0.71(0.38, 1.33)	0.75(0.40, 1.42)
**Elevated PP as reference**				
Normal PP	1.85(1.34, 2.55) ***	1.83(1.32, 2.52) ***	1.81(1.31, 2.50) ***	1.37(0.99, 1.90)
Stabilized PP	3.07(2.12, 4.46) ***	2.96(2.04, 4.29) ***	2.98(2.06, 4.33) ***	2.11(1.44, 3.08) ***
Excluding 192 participants screened as moderate or severe cognitive impairment at the first visit				
**Normal PP as reference**				
Stabilized PP	1.68(1.37, 2.06) ***	1.63(1.33, 2.00) ***	1.66(1.35, 2.04) ***	1.52(1.23, 1.87) ***
Elevated PP	0.56(0.41, 0.76) ***	0.56(0.41, 0.77) ***	0.57(0.42, 0.78) ***	0.71(0.52, 0.97) *
Persistently high PP	0.68(0.39, 1.18)	0.70(0.41, 1.22)	0.72(0.42, 1.25)	0.88(0.50, 1.52)
**Persistently high PP as reference**				
Stabilized PP	2.47(1.39, 4.39) **	2.32(1.30, 4.14) **	2.30(1.29, 4.10) **	1.74(0.97, 3.12)
Elevated PP	0.82(0.44, 1.53)	0.80(0.43, 1.50)	0.79(0.42, 1.47)	0.81(0.43, 1.52)
**Elevated PP as reference**				
Normal PP	1.79(1.32, 2.44) ***	1.77(1.30, 2.42) ***	1.76(1.29, 2.40) ***	1.41(1.03, 1.94) *
Stabilized PP	3.01(2.11, 4.31) ***	2.89(2.02, 4.15) ***	2.92(2.04, 4.18) ***	2.15(1.49, 3.09) ***

PP, pulse pressure. Hazard ratios (95% confidence intervals) are presented. Model 1 was adjusted for no covariates. Model 2 was adjusted for age, gender, ethnic group, education, primary occupation before retirement, average household income, and place of residence. Model 3 was adjusted for model 2 plus smoking, alcohol use, regular exercise, sleep quality, sleep

duration, and living alone. Model 4 was adjusted for model 3 plus heart rate, body mass index, hypertension, diabetes, heart disease, cerebrovascular disease, respiratory disease, and cancer. $^{*}P < 0.05$, $^{**}P < 0.01$, $^{***}P < 0.001$.
